# Daily emollient during infancy for prevention of eczema: the BEEP randomised controlled trial

**DOI:** 10.1016/S0140-6736(19)32984-8

**Published:** 2020-03-21

**Authors:** Joanne R Chalmers, Rachel H Haines, Lucy E Bradshaw, Alan A Montgomery, Kim S Thomas, Sara J Brown, Matthew J Ridd, Sandra Lawton, Eric L Simpson, Michael J Cork, Tracey H Sach, Carsten Flohr, Eleanor J Mitchell, Richard Swinden, Stella Tarr, Susan Davies-Jones, Nicola Jay, Maeve M Kelleher, Michael R Perkin, Robert J Boyle, Hywel C Williams, BEEP study team

**Affiliations:** a Centre of Evidence Based Dermatology, University of Nottingham, Nottingham, UK; b Nottingham Clinical Trials Unit, University of Nottingham, Nottingham, UK; c Skin Research Group, School of Medicine, University of Dundee, Dundee, UK; d Department of Dermatology, Ninewells Hospital and Medical School, Dundee, UK; e Population Health Sciences, University of Bristol, Bristol, UK; f Rotherham NHS Foundation Trust, UK; g Department of Dermatology, Oregon Health and Science University, Portland, OR, USA; h Sheffield Dermatology Research, Department of Infection and Immunity, University of Sheffield, Sheffield, UK; i Health Economics Group, Norwich Medical School, University of East Anglia, Norwich Research Park, Norwich, UK; j Unit for Population-Based Dermatology Research, St John's Institute of Dermatology, Guy's & St Thomas' NHS Foundation Trust and King's College London, London, UK; k Sheffield Children's Hospital, Sheffield, UK; l National Heart and Lung Institute, Imperial College London, London, UK; m St George's, University of London, London, UK

## Abstract

**Background:**

Skin barrier dysfunction precedes eczema development. We tested whether daily use of emollient in the first year could prevent eczema in high-risk children.

**Methods:**

We did a multicentre, pragmatic, parallel-group, randomised controlled trial in 12 hospitals and four primary care sites across the UK. Families were approached via antenatal or postnatal services for recruitment of term infants (at least 37 weeks' gestation) at high risk of developing eczema (ie, at least one first-degree relative with parent-reported eczema, allergic rhinitis, or asthma, diagnosed by a doctor). Term newborns with a family history of atopic disease were randomly assigned (1:1) to application of emollient daily (either Diprobase cream or DoubleBase gel) for the first year plus standard skin-care advice (emollient group) or standard skin-care advice only (control group). The randomisation schedule was created using computer-generated code (stratified by recruiting centre and number of first-degree relatives with atopic disease) and participants were assigned to groups using an internet-based randomisation system. The primary outcome was eczema at age 2 years (defined by UK working party criteria) with analysis as randomised regardless of adherence to allocation for participants with outcome data collected, and adjusting for stratification variables. This trial is registered with ISRCTN, ISRCTN21528841. Data collection for long-term follow-up is ongoing, but the trial is closed to recruitment.

**Findings:**

1394 newborns were randomly assigned to study groups between Nov 19, 2014, and Nov 18, 2016; 693 were assigned to the emollient group and 701 to the control group. Adherence in the emollient group was 88% (466 of 532) at 3 months, 82% (427 of 519) at 6 months, and 74% (375 of 506) at 12 months in those with complete questionnaire data. At age 2 years, eczema was present in 139 (23%) of 598 infants with outcome data collected in the emollient group and 150 (25%) of 612 infants in the control group (adjusted relative risk 0·95 [95% CI 0·78 to 1·16], p=0·61; adjusted risk difference –1·2% [–5·9 to 3·6]). Other eczema definitions supported the results of the primary analysis. Mean number of skin infections per child in year 1 was 0·23 (SD 0·68) in the emollient group versus 0·15 (0·46) in the control group; adjusted incidence rate ratio 1·55 (95% CI 1·15 to 2·09).

**Interpretation:**

We found no evidence that daily emollient during the first year of life prevents eczema in high-risk children and some evidence to suggest an increased risk of skin infections. Our study shows that families with eczema, asthma, or allergic rhinitis should not use daily emollients to try and prevent eczema in their newborn.

**Funding:**

National Institute for Health Research Health Technology Assessment.

## Introduction

Eczema (additionally termed atopic dermatitis or atopic eczema) affects about one in five children,^1^ is increasing in prevalence, and confers a high disease burden for individuals and their carers.^2^ Eczema usually starts in infancy, and persistence into adulthood is common.^3^ Children with eczema are more likely to develop other atopic conditions including food allergies, asthma, and allergic rhinitis.^4^ Because eczema usually precedes the development of food allergy, and early onset eczema is strongly associated with food allergy, prevention of eczema could prevent the development of food allergy.^5^, ^6^

Eczema is associated with loss-of-function mutations in *FLG,* the gene encoding filaggrin—a multi-functional protein that contributes to skin barrier integrity.^7^ This suggests that an impaired skin barrier could be a key defect in eczema development.^7^, ^8^ Sensitisation to food allergens can occur via a defective skin barrier.^9^, ^10^, ^11^, ^12^, ^13^, ^14^ Several observations support the rationale for emollients as a primary prevention intervention for eczema. Skin barrier dysfunction is apparent soon after birth and precedes eczema development, providing an opportunity for strategies to improve skin hydration and barrier function.^15^, ^16^ Emollients render the skin less susceptible to irritants such as soaps and detergents that could initiate eczema, can reduce percutaneous sensitisation by food antigens,^8^, ^15^ and can prevent flares of eczema (secondary prevention).^17^ Two small randomised pilot trials provided evidence that eczema prevention through emollients might be possible.^13^, ^14^ One study^13^ of 124 infants at high risk of eczema born in the UK and the USA showed that 22% of infants advised to use daily emollients developed eczema by age 6 months compared with 43% in controls (relative risk [RR] 0·50, 95% CI 0·28–0·90). The second study^14^ included 118 high-risk infants in Japan and showed that 32% of infants in the intervention group had eczema after 32 weeks versus 47% in the control group (hazard ratio 0·48, 95% CI 0·27–0·86). These strong efficacy signals from two small studies underpinned the decision of our funders to support a large-scale pragmatic trial to assess whether this intervention was effective when tested in normal practice.

**Research in context**

**Evidence before this study**

Genetic discoveries suggest that enhancing the skin barrier from birth might prevent eczema. In 2014, our pilot trial of 124 infants born to high-risk families suggested that daily emollients used for 6 months after birth could reduce development of eczema by around 50%. A similar study of 118 neonates in Japan found similar results with a 32% reduction in eczema at 32 weeks. We therefore designed the barrierenhancement for eczema prevention (BEEP) study to determine the effectiveness of advice to use emollients daily for the first year of life to prevent eczema in infants born to high-risk families. We searched the Centre of Evidence-Based Dermatology maps of systemic reviews of eczema prevention and Cochrane Central Register of Controlled Trials, MEDLINE, and online trial registries from Jan 1, 2000, until Aug 25, 2019, with no language restrictions, using synonyms for trials: “randomized controlled trial”, “controlled clinical trial”, “randomized”, “placebo”, “randomly”, “trial”; and for eczema: “dermatitis” or “eczema” or “neurodermatitis” or “besnier” and “prevention”.

We found 102 systematic reviews on eczema prevention but none dealing with barrier enhancement. Two randomised controlled trials of emollients for prevention of eczema have been published since our search: one from Japan including 459 infants (which tested Locobase REPAIR cream, Daiichi Sankyo, Japan) and the second from the USA including 100 infants (which tested Cetaphil Restoraderm, Galderma Laboratories, TX, USA). These two studies used ceramide-containing emollients, neither of which showed any significant preventive effect against development of eczema. Several additional ongoing trials investigating skin barrier interventions for eczema prevention have been identified, one of which (PreventADALL) is reported in this issue.

**Added value of this study**

The BEEP study of 1394 infants, the first large randomised controlled trial to specifically investigate whether emollients could prevent eczema, found no evidence to support the hypothesis that advice to use daily emollients can prevent eczema (primary outcome), food allergy, sensitisation, allergic rhinitis, or wheezing (secondary outcomes), and some evidence to suggest an increase in skin infections in those using emollients. Given the demanding nature of asking parents to apply emollients to the whole body for 12 months, we were pleased with reported adherence proportions of 88%, 82%, and 74% at 3 months, 6 months, and 12 months respectively.

**Implications of all the available evidence**

Since the publication of the two small efficacy studies, health-care professionals have started to recommend emollient for the primary prevention of eczema in high-risk families. This large, pragmatic study suggests that emollients used in this way do not prevent eczema and might be associated with harm, and that such practices should be stopped unless new evidence suggests otherwise. Knowledge of the neonatal skin barrier and optimal emollient formulation has progressed since the BEEP study was initiated, so new products might potentially exert a protective effect, which could be enhanced if accompanied by additional measures such as soft water and avoidance of soap.

Simple emollient formulations are inexpensive, widely available, and used extensively for treating eczema, and if effective in preventing eczema, could represent a breakthrough in reducing eczema incidence globally. We undertook this large, pragmatic trial to test the hypothesis that emollient use in the first year of life in high-risk infants (ie, with a family history of atopic disease) can prevent eczema and other atopic diseases including food allergy.

## Methods

### Study design and participants

This was a multicentre, two-arm, parallel-group, randomised controlled trial that was done at 12 hospitals and four primary care sites across the UK (appendix p 3). Families were approached largely via antenatal or postnatal services, by invitation letters from their general practitioners (GP), and through posters describing the study in hospitals and the community. Term infants (at least 37 weeks' gestation) at high risk of developing eczema (ie, at least one first-degree relative with parent-reported eczema, allergic rhinitis, or asthma diagnosed by a doctor) were included. Other inclusion criteria were mother aged 16 years or older, and the consenting adult had to have the ability to understand English. Exclusion criteria were as follows: preterm birth (birth before 37 weeks' gestation); a sibling (including twin) randomly assigned in the trial; a severe widespread skin condition that would make detection or assessment of eczema difficult; a serious health issue that would make it difficult for the family to take part in the trial; and a condition that would make the use of emollient inadvisable. Screening was usually done during the third trimester or shortly after delivery, and most families chose for this screening to take place in the family home.

Informed consent was obtained from mothers during pregnancy, or from the mother, father, or guardian after delivery. The trial was overseen by an independent Trial Steering Committee (appendix p3) and approved by the West Midlands Ethics Committee, UK (14/WM/0162). The study was sponsored by the University of Nottingham, coordinated by the Nottingham Clinical Trials Unit (CTU), and funded by the UK National Institute for Health Research (NIHR) Health Technology Assessment Programme. Supplementary funding was obtained for inclusion of food allergy outcomes and skin prick tests subsequent to study initiation, which was provided by Goldman Sachs Gives and Sheffield Children's Hospital Charity. The full protocol and changes made after the trial started are available online on the trial website and in the appendix (p 10). A protocol summary was published previously.^18^

### Randomisation and masking

Infants were randomly assigned (1:1) to receive either emollient and best practice skin-care advice (emollient group) or best practice skin-care advice only (control group). Infants were randomly assigned to a group within a maximum of 21 days after delivery, and randomisation was stratified by recruiting centre and number of first-degree relatives with atopic disease (1, 2, or >2). The randomisation schedule was created by the CTU using computer-generated pseudo-random code with permuted blocks of randomly varying size. The sequence was known only to the programmer until database lock. Research nurses randomly assigned participants to groups using a secure, internet-based randomisation system developed and maintained by the CTU. Parents were informed of their child's allocation by staff at the CTU. Research nurses doing skin examinations, skin prick testing, food challenges, or making food allergy decisions, and the statistician, were masked to treatment allocation during the study. Interim follow-up at 2 weeks and at 3, 6, 12, and 18 months was done by CTU staff to maintain research nurse masking. Participating families were not masked.

### Procedures

Families in the intervention group could choose between Doublebase Gel (Dermal Laboratories, Herts, UK) or Diprobase Cream (Bayer, Berks, UK). They were sent an initial package containing both emollients and parents specified on their resupply instructions which of the two emollients they wished to receive. Parents were advised to apply emollient to their child at least once daily to the whole body (excluding the scalp) until the child reached 1 year of age. They were also advised to apply emollient after every bath, even if they had already applied the emollient that day. Daily application was advised to encourage regular use of emollient several times a week, but because the study was designed to reflect how the intervention might be delivered in normal practice, no prompts or reminders were sent to parents. Parents were advised to stop applying emollients when their child reached 1 year of age, and no further emollients were supplied after this point.

Both groups received advice on general skin care in booklet and video format at the time of randomisation (appendix pp 11–21). The skin care guidance provided advice to use mild cleansers and shampoos specifically formulated for infants, and to avoid soap, bubble bath, and baby wipes.^19^ The guidance given to those in the emollient group also showed parents how to apply emollients correctly by dotting over the skin and using gentle downward strokes rather than rubbing in and contained warnings about the skin being slippery after application and the need to clean up spillages from the floor to avoid slipping. Parents were advised to seek medical advice as they would normally do if their child developed skin problems.

Follow-up was at 2 weeks (by telephone) and at 3, 6, 12, and 18 months (online or postal questionnaire). At the 2-year follow-up visit, the masked research nurse did the skin examination, saliva sample collection, skin prick testing, and provided the questionnaires, usually in the family home. In cases for whom a visit was not possible, data were collected by telephone, email, SMS, postal questionnaire, or from the GP. Methods for saliva collection, DNA extraction, and skin prick testing were published previously.^18^, ^20^ DNA samples were genotyped for the four most common *FLG* null mutations in the white European population (2282del4, R501X, S3247X, and R2447X).^21^ Participants who had a positive skin-prick test or a history suggestive of food allergy and in whom further investigation was required for a diagnosis of food allergy to be made were invited for a supervised oral food challenge. Food challenges were done at two hospitals by experienced allergy nurses masked to treatment allocation, following standard procedures.

### Outcomes

The primary outcome was diagnosis of eczema over the past year (defined by the UK working party refinement of the Hanifin and Rajka diagnostic criteria for eczema) assessed by research nurses masked to treatment allocation at age 2 years.^22^ This timepoint of 1 year after the intervention ended was chosen to ensure that the emollient had a genuine and lasting protective effect on eczema incidence as opposed to masking the emergence of mild eczema that might have occurred because of emollient use during year 1.^23^, ^24^

Secondary eczema outcomes were other eczema definitions—ie, presence of eczema between birth and 2 years of age (assessed by any parental report of a clinical diagnosis of eczema [up to 2 years] and parent completion of UK working party criteria at 1 and 2 years), presence of visible eczema at 2 years recorded by a nurse who was masked to treatment allocation; time to onset of eczema (based on first parent report of clinician diagnosis and time of first topical corticosteroid or immunosuppressant prescription); clinician-reported and patient-reported severity of eczema (Eczema Area and Severity Index [EASI] at 2 years and Patient-Oriented Eczema Measure [POEM] at 1 and 2 years). Other secondary outcomes were presence of other allergic diseases (ie, parent-reported wheezing and allergic rhinitis [between 1 and 2 years]; allergic sensitisation [masked skin prick tests] to milk, egg, peanut, cat dander, grass pollen, or dust mite at 2 years; parent-reported food allergy and parental report of clinical diagnosis of food allergy at 1 and 2 years; and allergy to milk, egg, or peanut at 2 years confirmed either by oral food challenge or for cases in which no oral food challenge was done, an expert allergy panel masked to treatment allocation). The expert panel decisions were made using a validated algorithm adapted from the EAT trial, which incorporates all available data including skin-prick test results, previous reaction history, frequency of food ingestion, and allergy tests done outside the trial.^25^ Safety outcomes were parent-reported skin infections (parents were asked what the doctor called the infection) and emollient-related infant slippages during the intervention period (year 1).

Analysis of health economic outcomes is underway and will be published separately. This will include analysis of health-care resource use at 3, 6, 12, 18, and 24 months, and cost-effectiveness and cost–utility at 24 months.

Quality of life was measured by Child Health Utility (CHU-9D) at 2 years to estimate quality-adjusted life-years (QALYs) in infants, and by EQ-5D-5L for parents at baseline and 2 years to estimate parental QALYs.

Adherence was captured at each questionnaire timepoint during year 1 (3, 6, and 12 months) by asking parents about emollient use since the last questionnaire (appendix p 4), and was defined in the protocol as satisfactory in the intervention group if emollients were applied at least 3–4 times per week to most of the child's body (defined as at least two of face and neck, arms and legs, or trunk). A similar definition was used for contamination in the control group.

Analysis and publication of data for the 2-year primary outcome timepoint were preplanned. Data collection for long-term outcomes at 3, 4, and 5 years is ongoing, but the trial is closed to recruitment of new participants.

### Statistical analysis

The trial was designed to detect a relative reduction of 30% in eczema at the 5% significance level (two-sided) with 90% power based on an expected rate of eczema of 30% in the control group and 20% attrition, resulting in a sample size of 1282.^18^ Quicker than expected recruitment prompted a review by the Trial Steering Committee (August, 2016), who permitted all pregnant mothers who had already given consent by that point to be randomly assigned to a study group upon the birth of the baby, allowing for a maximum recruitment total of 1400.

We analysed participants as randomised regardless of adherence with allocation and using observed data. The adjusted RR and difference in risk for the primary outcome were estimated using Generalised Estimating Equations with the Binomial family and log/identity link respectively, with an exchangeable correlation matrix to account for randomisation being stratified by centre and number of immediate family members with atopic disease (1, 2, or >2) included as a covariate. Sensitivity analyses were done using multiple imputation for missing data to include all participants in the analysis, split according to method of data collection (in person or by telephone, email, SMS, or post) and actual emollient use (complier average causal effect [CACE] accounting for emollient use in both groups). Analyses of secondary and safety outcomes used appropriate regression models and adjusted for stratification variables. Subgroup analyses for the primary outcome (diagnosis of eczema) and the secondary outcome of confirmed food allergy were done by including an interaction term in the analysis model for *FLG* genotype, number of first-degree relatives with atopic disease, and number of first-degree relatives with eczema. Additional subgroup analyses for the primary outcome for season of birth, water hardness in the home, and parent-reported probiotic supplements during pregnancy were in the statistical analysis plan (SAP) but not the protocol. Further details are presented in the appendix and SAP, finalised before the database lock. Analyses were done with Stata version 15.1. The trial was registered at the ISRCTN registry before initiation of recruitment, ISRCTN21528841.

### Role of the funding source

The main funder (NIHR Health Technology Assessment) was involved in refining the trial design through the funding peer review process, but had no role in data collection, data analysis, data interpretation, or writing of the report. The funders of the food allergy outcomes and skin prick tests (Goldman Sachs Gives and Sheffield Children's Hospital Research Fund) had no role in the study design, data collection, data analysis, data interpretation, or writing of the manuscript. HCW, AAM, and LEB had full access to all the data in the study, and HCW had final responsibility for the decision to submit for publication.

## Results

**Figure 1:**
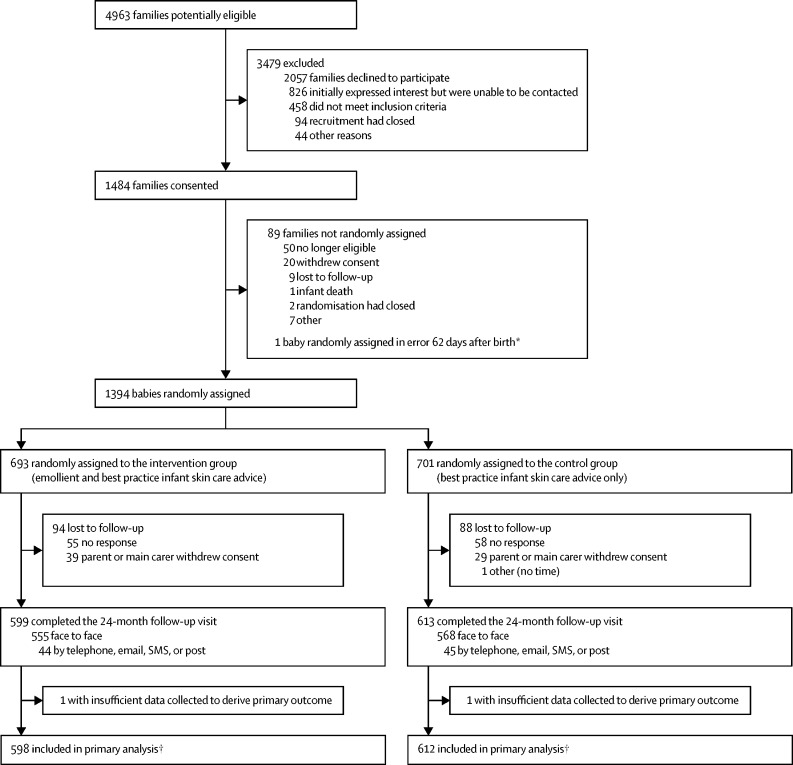
Trial profile *One family was randomly assigned in error at 62 days after birth and so was not included further. The family was not informed of the randomisation, not sent any intervention, and was not contacted for any follow-up. †A sensitivity analysis including all participants was also done using multiple imputation for missing data.

Between Nov 19, 2014, and Nov 18, 2016, 4963 families were assessed for eligibility at 12 hospitals and four general practice sites in the UK. 1394 babies were randomly assigned (1:1) to study groups (693 to the emollient group and 701 to the control group; figure 1). Baseline characteristics were balanced across groups (table 1). Accidental unmasking of outcome assessors to treatment allocation occurred for 3% of families at the 2-year visits (41 of 1190 participants who completed follow-up in person, or via telephone, text, or email; 30 in the emollient group and 11 in the control group). 509 families in the emollient group responded to a telephone call to check they had received the skin-care pack and emollients, and to collect data on the date they started applying the emollient to the infant. The median age of those in the emollient group when starting emollient was 11 days (IQR 7–17; n=509), and 452 (89%) of 509 had started applying emollient by day 21. Of families in the emollient group with complete questionnaire data on adherence at each timepoint, 466 (88%) of 532 had satisfactory adherence at 3 months, 427 (82%) of 519 at 6 months, and 375 (74%) of 506 at 12 months. 70% (311 of 442) of families with complete questionnaire data were classed as having satisfactory adherence at all timepoints during the first year. Using a highly conservative estimate that assumed 100% of those with no questionnaire data on adherence (ie, did not complete the questionnaires at 3, 6, or 12 months) did not apply the emollient, the proportion of families in the emollient group classed as having satisfactory adherence was estimated to be 51% (appendix p 32). On the days families used the intervention, most families chose to apply the emollient once a day (422 [79%] of 532 families at 3 months, 382 [74%] of 517 at 6 months, and 362 [72%] of 506 at 12 months). 93 (17%) of 532 families at 3 months, 104 (20%) of 517 families at 6 months, and 94 (19%) of 506 at 12 months chose to apply the emollient twice a day or more. Most participating families, with 479 (90%) of 533 at 3 months, 460 (89%) of 519 at 6 months, and 425 (84%) of 508 at 12 months, applied emollient to the arms, legs, and trunk. Additionally, most reported they usually applied emollient after a bath: 89% (471 of 532) at 3 months, 85% (441 of 516) at 6 months, and 80% (406 of 508) at 12 months. A median of 5 (IQR 2–5) 500 g containers dispensed over the year equated to about 7 g emollient per day over the first year. No emollient was supplied to the control group, but self-directed use of emollients at least three times per week to most of the body (contamination) occurred in 18% (82 of 457) at 3 months, 17% (62 of 372) at 6 months, and 15% (49 of 324) at 12 months, excluding children with a parental reported doctor diagnosis of eczema and who were therefore likely to be using an emollient to treat their eczema (appendix p 31).

**Table 1:** Baseline characteristics

		**Emollient group (n=693)**	**Control group (n=701)**
Age of mother at randomisation (mean [SD])		31·7 (5·3)	31·5 (5·2)
Parental-reported number of first-degree relatives with atopic disease			
	1	254 (37%)	253 (36%)
	2	300 (43%)	296 (42%)
	3 or more	139 (20%)	152 (22%)
At least one first-degree relative with history of eczema (parent report of doctor diagnosis)		563 (81%)	580 (83%)
Mother has eczema or had a history of eczema (parent report of doctor diagnosis)		348 (50%)	372 (53%)
Singleton pregnancy*		690 (100%)	696 (99%)
Gestation at birth in weeks (median [IQR])		40 [39·1–40·9]	40 [39·0–40·9]
Ethnicity of mother			
	White	589 (85%)	601 (86%)
	Asian	45 (6%)	40 (6%)
	Black	31 (4%)	22 (3%)
	Other	28 (4%)	38 (5%)
Decile of English index of multiple deprivation 2015 (median [IQR])		6 (3–9)	6 (3–8)
No other children living in household at screening		275 (40%)	293 (42%)
Sex			
	Male infant	374 (54%)	359 (51%)
	Female infant	319 (46%)	342 (49%)
Vaginal delivery		482 (70%)	472 (67%)
Furry pets living in house at time of birth		295 (43%)	302 (43%)
Maternal antibiotics during pregnancy		210 (30%)	201 (29%)
Maternal probiotics during pregnancy (collected at 6 months)		33/511 (6%)	32/505 (6%)
*FLG* genotyping†			
Number of infants		402	414
	+/+ (no mutations)	339/402 (84%)	352/414 (85%)
	+/− (one *FLG* null mutation)	62/402 (15%)	60/414 (14%)
	−/– (two *FLG* null mutations)	1/402 (<1%)	2/414 (<1%)

Data presented are n (%) or n/N (%) unless otherwise specified. *FLG*=gene encoding filaggrin.

* Three cases in the emollient group and five cases in the control group were twin and higher-order pregnancies.

† *FLG* genotype obtained from saliva samples at 2-year visit for infants whose parents consented to this part of the study. Samples were tested for the four most prevalent *FLG* loss-of-function mutations in the white European population. Of the 816 children included in the analysis, 810 (400 in the emollient group and 410 in the control group) had both parents of white ethnicity and six (two in the emillient group and four in the control group) had parents not of white ethnicity, but were included in the analysis because an *FLG* null mutation was detected.

Most infants in both groups were bathed or showered at least every other day (6 months, 81% of emollient group and 78% of control group; 12 months, 87% of emollient group and 87% of control group; 24 months, 90% of emollient group and 91% of control group). A third of participating families (32% of emollient group, 31% of control group) used water only (up to 6 months) and half used baby-specific wash products during the first year. Washing practices were balanced across groups (appendix pp 35–36).

Eczema in the past 12 months (UK working party criteria) at age 2 years was present in 139 (23%) of 598 infants in the emollient group and in 150 (25%) of 612 in the control group (adjusted RR 0·95 [95% CI 0·78 to 1·16]; p=0·61; adjusted risk difference –1·2% [–5·9 to 3·6]; table 2). Sensitivity analyses were consistent with the primary analysis, by use of data from GP records for missing primary outcome data, imputed missing data, and CACE analysis to evaluate adherence (appendix pp 40–42). Subgroup analyses according to number of first-degree relatives with atopic disease or eczema, *FLG* genotype, season of birth, water hardness, and probiotic use found no evidence of an interaction (appendix pp 42, 44–45).

**Table 2:** Primary and secondary eczema outcomes

			**Emollient group**	**Control group**	**Adjusted relative risk (95% CI)**	**Adjusted difference in risk (95% CI)**
Diagnosis of eczema at age 2 years according to UK working party diagnostic criteria*			139/598 (23%)	150/612 (25%)	0·95 (0·78 to 1·16)	−1·2% (−5·9 to 3·6)
Secondary eczema outcomes						
	At age 2 years					
		Masked assessment of visible eczema at age 2 years	151/555 (27%)	149/568 (26%)	1·05 (0·86 to 1·27)	1·1% (−4·0 to 6·3)
		Parent report of a clinical diagnosis of eczema between birth and age 2 years	266/610 (44%)	282/616 (46%)	0·96 (0·85 to 1·08)	−2·0% (−7·5 to 3·6)
		Eczema according to UK working party diagnostic criteria (parent completion)	187/599 (31%)	195/612 (32%)	0·98 (0·83 to 1·16)	−0·5% (−5·7 to 4·8)
		Moderate, severe, or very severe eczema according to EASI	9/553 (2%)	10/567 (2%)	0·93 (0·38 to 2·27)	0·0% (−1·5 to 1·4)
		Moderate, severe, or very severe according to POEM	58/576 (10%)	51/595 (9%)	1·18 (0·82 to 1·68)	1·7% (−1·6 to 5·0)
	At age 1 year					
		Eczema according to UK working party diagnostic criteria (parent completion)	103/516 (20%)	107/527 (20%)	0·98 (0·77 to 1·25)	−0·3% (−5·1 to 4·6)
		Moderate, severe, or very severe according to POEM	52/512 (10%)	49/522 (9%)	1·09 (0·75 to 1·57)	1·0% (−2·5 to 4·6)

Data are n/N (%) unless otherwise specified. The adjusted relative risk and difference in risk are estimated using generalised estimating equations with the binomial family and log/identity link respectively, with an exchangeable correlation matrix to account for randomisation being stratified by centre and number of immediate family members with atopic disease (1, 2, or more than 2) included as a covariate. EASI=Eczema Area and Severity Index. POEM=Patient-Oriented Eczema Measure.

* p=0·61.

**Figure 2:**
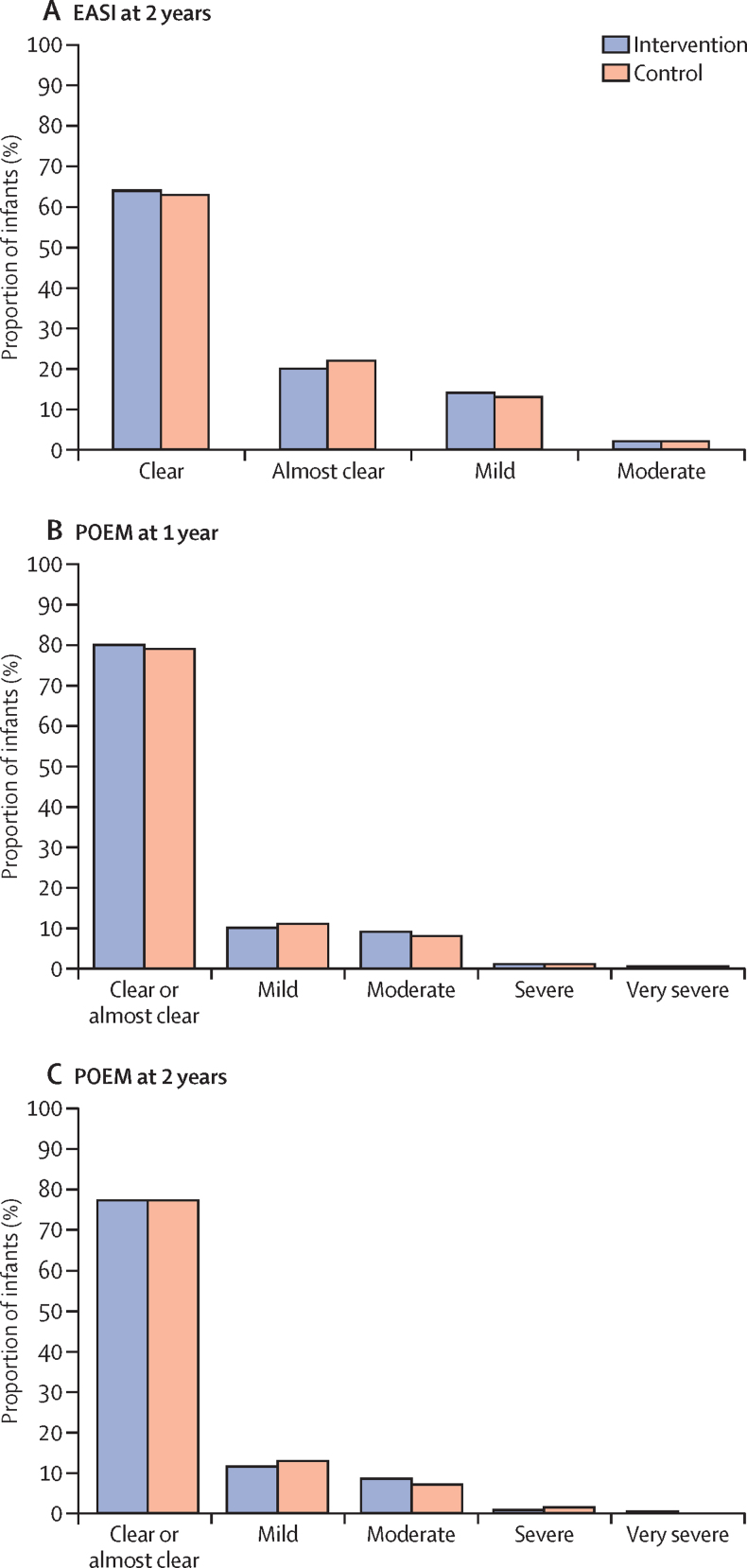
Severity of eczema assessed by clinician-reported signs measured by EASI (masked assessment) and parent-reported symptoms measured by POEM (A) Severity at 2 years measured by EASI (masked assessment by research nurse) based on categories in Leshem and colleagues.^26^ Parent-reported severity at 1 year (B) and 2 years (C) measured by POEM, based on categories in Charman and colleagues.^27^ These analyses include all infants for whom data were available, regardless of their eczema status. EASI=Eczema Area and Severity Index. POEM=Patient-Oriented Eczema Measure.

All other measures of eczema diagnosis were consistent with the primary outcome. There were no differences between groups in visible eczema at 2 years, parent report of a clinical diagnosis of eczema at 2 years, or parent completion of UK working party criteria at 1 and 2 years (table 2). Eczema severity assessed either by a masked assessment of clinician-reported signs (EASI)^26^ or parent-reported symptoms (POEM)^27^ was also similar between groups (figure 2), as was time to onset of eczema (appendix pp 47–50). Food allergies to milk, egg, or peanut were confirmed in 41 (7%) of 547 infants in the emollient group and 29 (5%) of 568 in the control group (adjusted RR 1·47, 95% CI 0·93–2·33). The largest difference was in the proportion of infants with confirmed food allergy to egg, with an adjusted RR of 1·56 (95% CI 0·92–2·65). Of the confirmed food allergy diagnoses, 30% (21 of 70; 15 in the emollient group and six in the control group) were made with the oral food challenge and 70% (49 of 70; 26 in the emollient group and 23 in the control group) were by use of the algorithm adapted from the EAT trial.^25^ The results of other measures of food allergy and food sensitisation were similar (appendix pp 59–61). The proportion of infants with allergic rhinitis, wheezing, and allergic sensitisation to cat dander, grass pollen, and dust mite was similar between groups (table 3). There were no differences in quality of life utility measures (CHU-9D and EQ-5D-5L) between the two groups (table 4).

**Table 3:** Secondary outcomes of confirmed food allergy, sensitisation to common food allergens, allergic rhinitis, and wheezing at age 2 years

	**Emollient group**	**Control group**	**Adjusted relative risk (95% CI)**	**Adjusted difference in risk (95% CI)**
**Confirmed food allergy**				
Confirmed allergy to milk, egg, or peanut at age 2 years*	41/547 (7%)	29/568 (5%)	1·47 (0·93 to 2·33)	2·4% (−0·5 to 5·2)
Confirmed allergy to cow's milk at age 2 years†	9/571 (2%)	8/593 (1%)	1·17 (0·45 to 3·01)	0·2% (−1·2 to 1·6)
Confirmed allergy to egg at age 2 years	33/560 (6%)	22/581 (4%)	1·56 (0·92 to 2·65)	2·1% (−0·4 to 4·6)
Confirmed allergy to peanut at age 2 years	10/555 (2%)	8/572 (1%)	1·29 (0·51 to 3·25)	0·4% (−1·1 to 1·8)
**Sensitisation to food allergens**‡§				
Allergic sensitisation to milk, egg, or peanut at age 2 years	58/487 (12%)	44/498 (9%)	1·36 (0·94 to 1·95)	2·9% (−0·9 to 6·8)
Allergic sensitisation to milk at age 2 years	14/488 (3%)	11/498 (2%)	..	..
Allergic sensitisation to egg at age 2 years	43/490 (9%)	33/499 (7%)	..	..
Allergic sensitisation to peanut at age 2 years	18/490 (4%)	16/502 (3%)	..	..
**Sensitisation to other allergens**				
Allergic sensitisation to grass pollen, cat dander, or dust mite at age 2 years¶	50/492 (10%)	48/499 (10%)	1·07 (0·74 to 1·55)	0·9% (−2·8 to 4·5)
**Other allergies**				
Allergic rhinitis, parent report between age 1 and 2 years	174/572 (30%)	188/598 (31%)	0·97 (0·82 to 1·15)	−0·8% (−6·2 to 4·5)
Wheezing, parent report between age 1 and 2 years	197/572 (34%)	191/598 (32%)	1·07 (0·91 to 1·26)	2·5% (−2·9 to 7·9)

Data are n/N (%) unless otherwise specified.

* Food allergy was confirmed by oral food challenge interpreted by a masked allergy nurse using PRACTALL criteria in 21 of 70 participants, or using a validated algorithm adapted from the EAT trial interpreted by a masked expert allergy panel (MJC, NJ, MK, RJB) in 49 of 70.^25^

† Unadjusted relative risk and difference in risk reported for cow's milk. The model including stratification variables did not converge.

‡ ≥3 mm skin prick test to fresh milk, raw egg white, or commercial peanut extract.

§ Adjusted difference in risk and relative risk is not presented for individual allergens (specified in statistical analysis plan that between-group estimates would only be calculated for allergens grouped together). Allergic sensitisation to individual allergens tested is presented in the appendix (p 59).

¶ ≥3 mm skin prick test to at least one of commercial grass pollen, cat dander, or dust mite extract.

**Table 4:** Secondary outcome of quality of life

	**Emollient group**	**Control group**	**Unadjusted difference in means (95% CI)**
CHU-9D at age 2 years*	0·935 (0·070)	0·934 (0·066)	0·001 (−0·007 to 0·009)
EQ-5D-5L parent health-related quality of life at baseline†	0·856 (0·151)	0·852 (0·158)	..
EQ-5D-5L parent health-related quality of life at age 2 years‡	0·921 (0·142)	0·919 (0·130)	0·002 (−0·013 to 0·018)

Data are mean (SD) unless otherwise specified. CHU-9D scores range from 0·33 to 1, with higher scores indicating better quality of life. EQ-5D-5L scores range from −0·594 to 1, with higher scores indicating better quality of life. Of those with data at baseline and age 2 years, 95% were completed by the same responder. The baseline EQ-5D-5L questionnaire was sent to parents in the post shortly after randomisation. CHU-9D=Child Health Utility-9 Dimensions.

* n=573 in the emollient group and n=591 in the control group.

† n=496 in both the emollient group and the control group.

‡ n=573 in the emollient group and n=592 in the control group.

Parent-reported doctor-diagnosed skin infections during the first year occurred in 89 (15%) of 585 infants in the emollient group and in 67 (11%) of 589 in the control group. Impetigo and unspecified bacterial, viral, or fungal skin infections accounted for the majority and a full breakdown of the type of infection can be found in the appendix (p 62). The mean number of skin infections per child was 0·23 (SD 0·68) in the emollient group and 0·15 (0·46) in the control group (adjusted incidence rate ratio of 1·55, 95% CI 1·15–2·09; appendix p 62). Parent-reported infant slippages within an hour of applying emollients were rare and the frequency was similar between groups: 15 (3%) of 584 in the emollient group and 11 (2%) of 584 in the control group (adjusted RR 1·37, 95% CI 0·63–2·97). None of the slippages resulted in serious injury or admission to hospital.

## Discussion

In this multicentre, pragmatic, randomised controlled trial of high-risk infants, we did not find any evidence that regular emollient use for the first year of life can delay, suppress, or prevent eczema at age 2 years. The results for the primary outcome excluded our prespecified relative reduction of 30%. This finding was consistent regardless of how eczema was defined. There was no evidence that emollients reduced the risk of food allergy; a non-significant increase in food allergy in the emollient group was observed compared with controls. This was largely because of the higher number of participants in the emollient group being diagnosed with egg allergy. Furthermore, we observed an increase in parent-reported skin infections. Notably, we emphasise that our findings relate only to using emollients for preventing eczema, and not the use of emollients for treating eczema.

Strengths of this study include the large sample size and good rates of treatment adherence, high retention for the primary outcome, and low rates of contamination, particularly given that it was a long-term prevention study of healthy infants with little investigator contact. Our study tested an intervention that was acceptable to parents and the use of emollients in the study is likely to closely reflect how emollients are recommended and used in the community.^28^ Selection, detection, performance, and attrition biases are unlikely to explain the absence of a preventive effect. Eczema was measured using validated criteria, applied by trained researchers masked to the treatment intervention 1 year after use of the emollient had ceased. The primary outcome results were collected by masked researchers, although the diagnostic criteria do include questions that are answered by unmasked parents. Findings were supported by a masked evaluation of eczema that did not rely on parent reporting. Assessing the primary outcome at age 2 years excludes transient eczematous rashes that are common in the first year of life and ensures that any eczema present is not concealed by the application of emollient during the first year—issues that might have accounted for the protective effect of emollients seen in the two previous smaller pilot studies.

The study emollients were chosen because they are commonly used in the UK National Health Service and have a high degree of acceptability to parents, established during preparatory work. Both emollients are a basic formulation containing petrolatum and no ingredients known to have a detrimental effect on the skin barrier, in particular sodium lauryl sulphate. Mechanistic studies showed that these emollients were appropriate to use in such a trial, compared with aqueous cream, which has adverse effects on the skin barrier if used as a leave-on emollient.^29^, ^30^ We did not use a more complex emollient formulation—for example, one containing ingredients such as ceramides and pH modulators—because when the barrier enhancement for eczema prevention (BEEP) trial was designed, they were not generally available and had a much higher purchase cost. However, a more sophisticated emollient formulation might potentially have a protective effect.

Limitations of the study include a low uptake of the oral food challenges due to several factors. Skin prick tests and oral food challenges were added to the trial once it was underway, so parents were unaware these tests would be offered when they decided to take part. Added to this, many parents were unwilling or unable to travel to the oral food challenge centres, mainly because they had no concern about food allergy in their child or their child had a previously established clinical diagnosis of food allergy. There is also significant uncertainty about the food allergy outcomes, which is not surprising as the study was powered to detect plausible changes in eczema rather than food allergy, which has a much lower incidence. A further limitation was the lower than expected response to interim questionnaires through which adherence was assessed.

The lack of benefit of emollients for prevention of eczema seen in this study was unexpected, particularly when considering the strong signal observed in the previously published pilot trials, and has major implications for the primary prevention of atopic eczema and other diseases.^13^, ^14^ Adherence to the advice to apply emollients once a day might not have been sufficiently high enough to have an effect, or insufficient quantities might have been used during each application. Applying emollients all over the body daily for the first 12 months of life can be difficult to maintain for busy parents of healthy babies. However, data for those who completed questionnaires in which parents were asked about adherence to emollient use since the previous questionnaire showed that adherence was greater than 80% during the first 6 months, which was within our anticipated range, with most parents reporting applying emollient to the whole body. These adherence figures were similar to those seen in our pilot study.^13^ Although adherence decreased to 74% for months 6–12, this was a pragmatic study in which parents had little contact with the research team, reflecting how such a prevention strategy might be delivered in practice. Additionally, this reduction in emollient use was expected because of increased difficulty in regularly applying emollient to infants as they become more mobile as well as general parental fatigue with regards to the routine. There was a small amount of emollient use in the control group in those without any diagnosis of eczema, which could have partially masked any differences between the two groups. Emollients might have to be applied multiple times per day to exert a protective effect, or intervention for longer than a year might be required, but a more demanding schedule is more difficult to maintain or could be unacceptable to parents. Although the median duration between birth and start of emollient use was only 11 days, even earlier intervention might be required with emollient therapy started closer to birth. The provision of skin care advice to parents in both groups was unlikely to have altered any effect of the emollient, since the advice was based on best practice skin-care advice in the UK and was identical to that used in the pilot trial in which a difference between groups was observed. However, awareness of the importance of using better formulated wash products has increased in recent years.^13^

Although there was no significant difference in occurrence of food allergy between the two groups, any degree of increase in the emollient group was unexpected. The non-significant association between emollient intervention and increased food allergy was seen across several different measures, including objective tests of skin sensitisation. An increase in food allergy is plausible, through enhanced transfer and uptake of food antigens by emollient application, leading to epicutaneous sensitisation.^31^ An ongoing prospective individual patient data meta-analysis^32^ of at least nine similar studies including the PEBBLES trial (NCT03667651) and the PreventADALL trial^33^ will provide more evidence on any association between use of emollients from birth and the risk of developing food allergy. The evidence for increased skin infections with emollients was stronger and could be due to increased inoculation of pathogens on the infant skin during application of emollients,^34^ disturbance of the skin microbiome, or emollients making the skin more adhesive to bacteria.

Understanding of the properties of the barrier function of the skin in early life has increased and it is possible that other approaches to skin barrier enhancement based on new knowledge might have a preventive effect. These approaches might include using newly developed emollients with enhanced skin barrier properties, or a complex intervention that includes additional stringent measures such as low pH cleansers, infrequent washing, or softened water. Future studies of new candidate emollients or more burdensome skin-care regimens will first need to ensure acceptability to parents and consider the inclusion of behavioural support to ensure adherence for prolonged periods.

Our study does not support the use of emollients for preventing eczema in high-risk infants, a finding supported by PreventADALL, another large trial using a skin barrier enhancing intervention.^33^ Our data relate only to prevention of eczema and do not directly challenge the practice of using emollients as first-line treatment for eczema.^35^

## Data sharing

Data collected for the study, including de-identified individual participant data and a data dictionary defining each field in the set, will be made available to researchers who provide a methodologically sound proposal to the corresponding author with a signed data access agreement. The study protocol, statistical analysis plan and health economics analysis plan are available on the trial website and the NIHR journals library. All other related documents are available on request to Prof Hywel C Williams as chief investigator of the BEEP trial, at any point.
