# Supplementary material for: Daily emollient during infancy for prevention of eczema: the BEEP randomised controlled trial
Source: Lancet. 2020 Mar 21;395(10228):962–72. doi: 10.1016/S0140-6736(19)32984-8 (PMC7086156; doi:10.1016/S0140-6736(19)32984-8)
Supplement: Supplementary appendix [file mmc1.pdf]

THE LANCET

Supplementary appendix

This appendix formed part of the original submission and has been peer reviewed. We post it as supplied by the authors.

Supplement to: Chalmers JR, Haines RH, Bradshaw LE, et al. Daily emollient during infancy for prevention of eczema: the BEEP randomised controlled trial. *Lancet* 2020; published online Feb 19. http://dx.doi.org/10.1016/S0140-6736(19)32984-8.

Supplementary Material

Contents

Study information ... 3

Recruiting sites and staff ... 3

Trial steering committee members ... 3

Further details on statistical methods ... 4

Assessment of compliance and contamination ... 4

Summary of compliance and contamination over the first year ... 5

Sensitivity analysis ... 5

Further details on multiple imputation ... 6

Further details on estimating the complier average causal effect (CACE) ... 7

Primary outcome definition – UK Working Party Diagnostic Criteria (under 4's version) ... 7

Changes to protocol after start of trial ... 10

Skin care advice ... 11

Intervention group ... 11

Control group ... 19

Study quality summaries ... 22

Results ... 35

References ... 64

Table S1: Changes to protocol after start of trial 10
Table S2: Randomisation by recruiting centre 22
Table S3: Questionnaire completion rates at 3, 6, 12 and 18 months 23
Table S4: 2 year visit completion rate 24
Table S5: Genetic study (*FLG)* completion rate 25
Table S6: Skin prick test completion rate 26
Table S7: Food allergy assessment rate 27
Table S8: Adherence; parent report of use of study emollient in the intervention group during the first year 28
Table S9: Contamination; parent report of (non-study) self-directed emollient use in the control group during the first year 31
Table S10: Summary of adherence and contamination during the first year 32
Table S11: List of Protocol deviations (as reported by research nurses on the eCRF) not relating to food allergy assessment process 33
Table S12: List of Protocol deviations (as reported by research nurses on the eCRF) relating to food allergy assessment process 34
Table S13: Frequency of bathing/showering of children 35
Table S14: Other post-randomisation practices 37
Table S15. Timing of introduction of allergenic foods asked at 24 months 39
Table S16: Sensitivity analyses for primary outcome 40
Table S17: Complier average causal effect for primary outcome of eczema between the age of 1 and 2 years .. 42
Table S18: Subgroup analyses for primary outcome of eczema between the age of 1 and 2 years 43
Table S19: Eczema severity (POEM and EASI) 46
Table S20: Time to onset of eczema based on first parental report of clinical diagnosis of eczema 47
Table S21: Time to onset of eczema based on first parental report of a topical corticosteroid or immunosuppresant prescription for eczema AND a parental report of a clinical diagnosis of eczema. 49
Table S22: Confirmed food allergy at 2 years 51
Table S23: Sensitivity analysis for confirmed food allergy at 2 years, including panel consensus decisions of unclear (food allergy possible or food allergy unlikely) 52
Table S24: Sensitivity analysis for confirmed food allergy at 24 months to any of milk, egg or peanut using multiple imputation for missing outcomes 53
Table S25: Summary of compliance/contamination and confirmed food allergy at 24 months to any of milk, egg or peanut 54
Table S26: Complier average causal effect for confirmed food allergy at 2 years to any of milk, egg or peanut 55
Table S27: Subgroup analyses for confirmed food allergy at 2 years to any of milk, egg or peanut 56
Table S28: Rate of allergic sensitisation at 2 years 59
Table S29: Parent reported food allergy 60
Table S30: Sensitivity analysis for parental report of clinical diagnosis of food allergy at 2 years 61
Table S31: Parent-reported skin infections during the first year (as randomised) 62
Table S32: Infant slippage incidents within an hour of applying skin care products to the baby's skin during the first year (as randomised) 63

Figure S1: Flowchart taken from study protocol indicating the process for food allergy assessments 8
Figure S2: Detailed algorithm used to classify food allergy status for participants invited to oral food challenges but did not attend 9
Figure S3: Forest plot of adjusted relative risk for primary outcome with 95% confidence intervals in each subgroup 44
Figure S4: Forest plot of adjusted risk difference for primary outcome with 95% confidence intervals in each subgroup 45
Figure S5: Time to onset of eczema based on first parental report of a clinical diagnosis of eczema 48
Figure S6: Time to onset of eczema based on first parental report of a topical corticosteroid and/or immunosuppressant prescription for eczema AND a parental report of a clinical diagnosis of eczema. 50
Figure S7: Forest plot of adjusted relative risk for confirmed food allergy at 24 months to any of milk, egg or peanut with 95% confidence intervals in each subgroup 57
Figure S8: Forest plot of adjusted risk difference for confirmed food allergy at 24 months to any of milk, egg or peanut with 95% confidence intervals in each subgroup 58

Study information

Recruiting sites and staff

Nottingham University Hospitals NHS Trust: Professor Hywel C. Williams (PI), Susan Davies-Jones, Professor Jim Thornton; Barbara Maston, Victoria Maddox, Faye Shelton, Catherine Thorne

Portsmouth Hospitals NHS Trust: Dr. Bronwyn Hughes (PI), Andrew Gribbin, Sharon McCready, Zoe Garner, Amanda Hungate, Emma Glasspool, Rachel Watson,;

Harrogate and District NHS Foundation Trust: Dr. Alison Layton (PI), Louise Wills, Elizabeth Marshall, Joyce Guy, Christine Morgan;

Sherwood Forest Hospitals NHS Foundation Trust: Dr. Michael Yanney (PI), Caroline Moulds, Lisa Foster, Yvette Girvan, Victoria Moore, Andrea Palfreman;

Burton Hospitals NHS Foundation Trust: Dr. Mansoor Ahmed (PI), Stephanie Boswell, Claire Prince, Jane Radford, Clare Mewies, Claire Backhouse, Elizabeth Kemp;

Derby Hospitals NHS Foundation Trust: Dr. Adam Ferguson (PI), Elaine Coulborn, Melody McGregor, Coral Smith, Vanessa Unsworth, SallyAnn Bell, Jill Smith, Liane Hufton;

University Hospitals of Leicester NHS Trust: Dr. Karen Harman (PI), Dr. Ingrid Helbling, Suzanne Foxon, Simal Patel, Jackie Philps, Esther Rook;

York Teaching Hospital NHS Foundation Trust: Dr. Calum Lyon (PI), Anna Clayton, Jill Green, Jessica Scott, Richard Furnival, Samantha Roche, Holly Alcock, Sian Sturdy;

Sheffield Children's NHS Foundation Trust: Professor Michael Cork (PI) Heather Chisem, Hilary Rosser, Alyson Barber, Sarah Besley, Emma Steel, Sarah Senbeto, Pauline Bayliss, Carolyn Clark;

Imperial College Healthcare NHS Trust: Dr. Robert Boyle (PI), Anna Bosanquet, Batia Gourin;

Guy's and St Thomas' NHS Foundation Trust: Dr. Carsten Flohr (PI), Nikeeta Gurung, Annette Briley, Claire Singh, Rebecca Williams, Shelley Carter, Elodie Lawley;

Bristol: Dr. Matthew Ridd (PI), Kingsley Powell, Lyn Liddiard, Anna Gilberston.

Primary Care recruitment sites: Francis Grove Surgery: Dr. Katherine Broad (PI), Nina Walters, Sarah Buttinger, Rachel Joy; Streatham Common Practice: Dr. Kirsty Rankin (PI), Dr. Ruth Danson, Ellen Trendell; Clapham Park Group Practice: Dr. Mydhili Chellappah (PI), Dr. Dina Saleh; Park Group Practice: Dr. Mita Patel (PI), Jayshireen Singh

Trial steering committee members

- Sarah Meredith, Professor of Clinical Trials, University College London, (Chair);
- Angela Crook, Senior Statistician, University College London;
- Paula Beattie, Consultant Dermatologist, Queen's University, Belfast;
- Kirsty Logan, Research Fellow, King's College London;
- Emma Thomas, PPI Representative

Further details on statistical methods

Assessment of compliance and contamination

Compliance and contamination were assessed by parent recall since the last questionnaire. Parents were asked the following questions at each questionnaire timepoint (X = number of months since previous questionnaire):

Compliance (intervention group) at 3, 6 and 12 months (intervention period):

Over the last X months, how often have you usually used the moisturiser that we sent you on your baby?<br>(tick only one)	Everyday ☐<br>5 - 6 days per week ☐<br>3 - 4 days per week ☐<br>Once or twice a week ☐<br>Never ☐
In the last X months, where on your baby have you usually applied the moisturiser?<br>(tick all that apply)	Face/neck ☐<br>Arms/legs ☐<br>Trunk ☐
In the last X months, how many times a day have you usually applied the moisturiser?<br>(tick only one)	Once ☐<br>Twice ☐<br>More than Twice ☐

Contamination (control group) at 3, 6, 12 months (and both groups in all questionnaires after 12 months):

In the last X months, have you regularly applied any moisturisers to your baby's skin (except for treating nappy rash or cradle cap) or oil for baby massage?	Yes ☐ N<br>o ☐

If you answered 'yes' to the above question, please answer the following three questions:

Please tell us what have you used	____________________<br>____________________<br>____________________
In the last X months, how often have you usually applied these to your baby's skin?<br>(tick only one)	Everyday ☐<br>5 - 6 days per week ☐<br>3 - 4 days per week ☐<br>Once or twice a week ☐
In the last X months, where on your baby have you usually applied these moisturisers or oils?<br>(tick only one)	Over most or whole of the body ☐<br>Small patches or areas of the body only ☐

Summary of compliance and contamination over the first year

Compliance and contamination over the first year of life was described using an ordered categorical variable, as defined below:

Level of compliance in the intervention group/contamination in the control group	Criterion for compliance/contamination met at the following time points
Full	3, 6 and 12 months
Early onset application	3 months (with neither or only one of 6 or 12 months)
Late onset application	6 and/or 12 months (but not at 3 months)
None	Compliance/contamination criterion not met at any of 3, 6 or 12 months

Compliance in the intervention group at each time point defined as wide spread emollient use over the majority of the child's body at least three or more days per week. Majority of the child's body defined as at least 2 of the 3 body areas asked about on the questionnaire (face/neck, arms/legs or trunk).
Contamination at each time point in the control group defined as use of a moisturiser or oil at least three days per week over most or all of the child's body since the last questionnaire.

Compliance/contamination was summarised for the subset of participants with complete data on compliance/contamination (i.e. completed questionnaires at 3, 6 and 12 months) and for all participants using the assumptions for missing data described below.

1. Participants with no reported data on emollient/moisturiser use were categorised as not compliant in the intervention arm and not contaminated in the control arm.
2. If participants missed a questionnaire(s) and went onto complete a subsequent questionnaire, missing emollient/moisturiser use was based on the next subsequent observation carried backwards. For example if the 6 month questionnaire was missed for a participant in the intervention group and they reported being compliant at 12 months then it was assumed that they were also compliant at the 6 month time point. The rationale for this is that it is assumed that compliance is likely to decrease over time so this is conservative for the intervention group.
3. If participants completed questionnaires initially and miss later questionnaires (e.g. completed at 3, did not complete 6 or 12 or completed at 3 and 6, did not complete 12) then it was assumed that there was no compliance (intervention) /contamination (control) for the later missed questionnaires.
4. Categorisation of compliance/contamination over the first 12 months following randomisation for participants with missing emollient/moisturiser use data then proceeded according the definitions above.

Sensitivity analysis

The following sensitivity analyses for the primary outcome were performed:

- Analysis repeated including diagnosis of eczema data collected from GP records for participants with missing primary outcome data
- According to method of collection of the primary outcome data: between group estimates for the risk of eczema were calculated separately for outcomes collected during face to face visits and outcomes collected by telephone/email/SMS using Generalised Estimating Equations adjusting for stratification variables
- Analysis repeated replacing "visible flexural dermatitis" in the UKWP criteria with any visible dermatitis (EASI score of greater than zero) in the derivation of eczema between one and two years of age
- Using multiple imputation for missing primary outcome data (further details described below)
- Repeating the analysis assuming that all participants in the intervention group with missing primary outcome data were eczema free and all participants in the control group with missing data had eczema (i.e. best case scenario for intervention)

- Repeating the analysis assuming that all participants in the intervention group with missing primary outcome data had eczema and all participants in the control group with missing data were eczema free (i.e. worst case scenario).

In addition, to explore the effect of application of emollient in the first year of life in parents who would comply with the allocated treatment, the complier average causal effect (CACE) was estimated[2] (further details described below).

The following sensitivity analyses for the confirmed food allergy outcome (food allergy at 24 months to any of milk, egg or peanut) were performed:

- Repeating the analysis including panel decisions of "unclear – possible food allergy" as allergic and "unclear – food allergy unlikely" as not allergic

- Using multiple imputation for missing outcomes (further details described below)

In addition, to explore the effect of application of emollient in the first year of life in parents who would comply with the allocated treatment, the complier average causal effect (CACE) was also estimated for the confirmed food allergy outcome (further details described below).

Sensitivity analysis was also performed for parent report of clinical diagnosis of food allergy at 2 years outcome as this was unknown for children whose parents did not complete the 12 month questionnaire and reported that their child did not have a clinical diagnosis of food allergy between 12 and 24 months (as the child may have had a diagnosis between birth and 12 months). For these children, multiple imputation was used for the unknown information at 12 months. In addition for children with no 24 month follow-up, data collected from GPs on diagnosis of food allergy between 12 and 24 months was used in this sensitivity analysis.

Further details on multiple imputation
Multiple imputation was performed using chained equations.[3] The following variables were used in the imputation model:

- Allocated group

- Randomisation stratification variables: centre, number of immediate family members with atopic disease (one, two, three or more).

- Age of mother, number of other children in household at screening (none, one, two, three or more), any furry pets in the household at screening (yes/no) and baby sex (baseline variables identified as predictive of drop-out by examination only),

- variables used in subgroup analyses - number of *FLG* null mutations (none, one or two), the number of immediate family members with eczema (zero, one, two or more), water hardness (soft, moderate, hard, very hard), season of birth (spring, summer, autumn, winter) and regular use of probiotic supplements during pregnancy (yes/no).

- Summary of compliance and contamination in the first year of life as per the definitions above.

The following outcomes were imputed:

- Diagnosis of eczema in the last year at age 2 years (UK working party refinement of the Hanifin and Rajka diagnostic criteria, primary outcome)

- Parental report of a clinical diagnosis of eczema at 2 years

- Parental report of immediate allergy to cow's milk, egg or peanut at 2 years

- Allergic sensitisation to cow's milk, egg or peanut

- Food allergy to any of milk, egg or peanut at 2 years

Forty datasets were imputed and the results of the analyses on the imputed datasets were combined using Rubin rules for multiply imputed data. This analysis assumed that unobserved outcomes are missing at random and depend on observed characteristics but not the unobserved outcomes.

On checking the imputed values from the multiple imputation model above, it was observed that the percentage of children with each of the food allergy outcomes was higher in the imputed data than in the observed data. Therefore a simpler multiple imputation model including only allocated group and the randomisation stratification variables with 20 imputations was used to impute the primary outcome and the outcome of food allergy to milk, egg or peanut at 2 years to check the robustness of the results.

Further details on estimating the complier average causal effect (CACE)
CACE models were implemented as latent growth mixture models[1] with compliance/contamination status included as a training variable for estimating class membership. The CACE estimate is presented as an odds ratio and MPlus was used for the analysis.

Two separate odds ratios for the CACE were estimated based on the following definition of compliance:

(1) full compliance over the first year of life as per table A – participants in the control group who met the criteria for full contamination were considered as always-takers.

(2) compliance within the first three months (i.e. in the full compliance or early onset application categories in Table A) – participants in the control group who met the criteria for full or early onset contamination were considered as always-takers.

For children with incomplete data on compliance/contamination, the assumptions described above were used to categorise their compliance/contamination.

Primary outcome definition – UK Working Party Diagnostic Criteria (under 4's version)

Must have:

An itchy skin condition in the last year

Plus three or more of:

i. History of flexural or cheek involvement (ever)

ii. History of a generally dry skin (in the last year)

iii. History of atopic disease in a first degree relative [1]

iv. Visible flexural dermatitis as per photographic protocol (for under 4's)

[1] *Entry criteria for the trial*

Figure S1: Flowchart taken from study protocol indicating the process for food allergy assessments

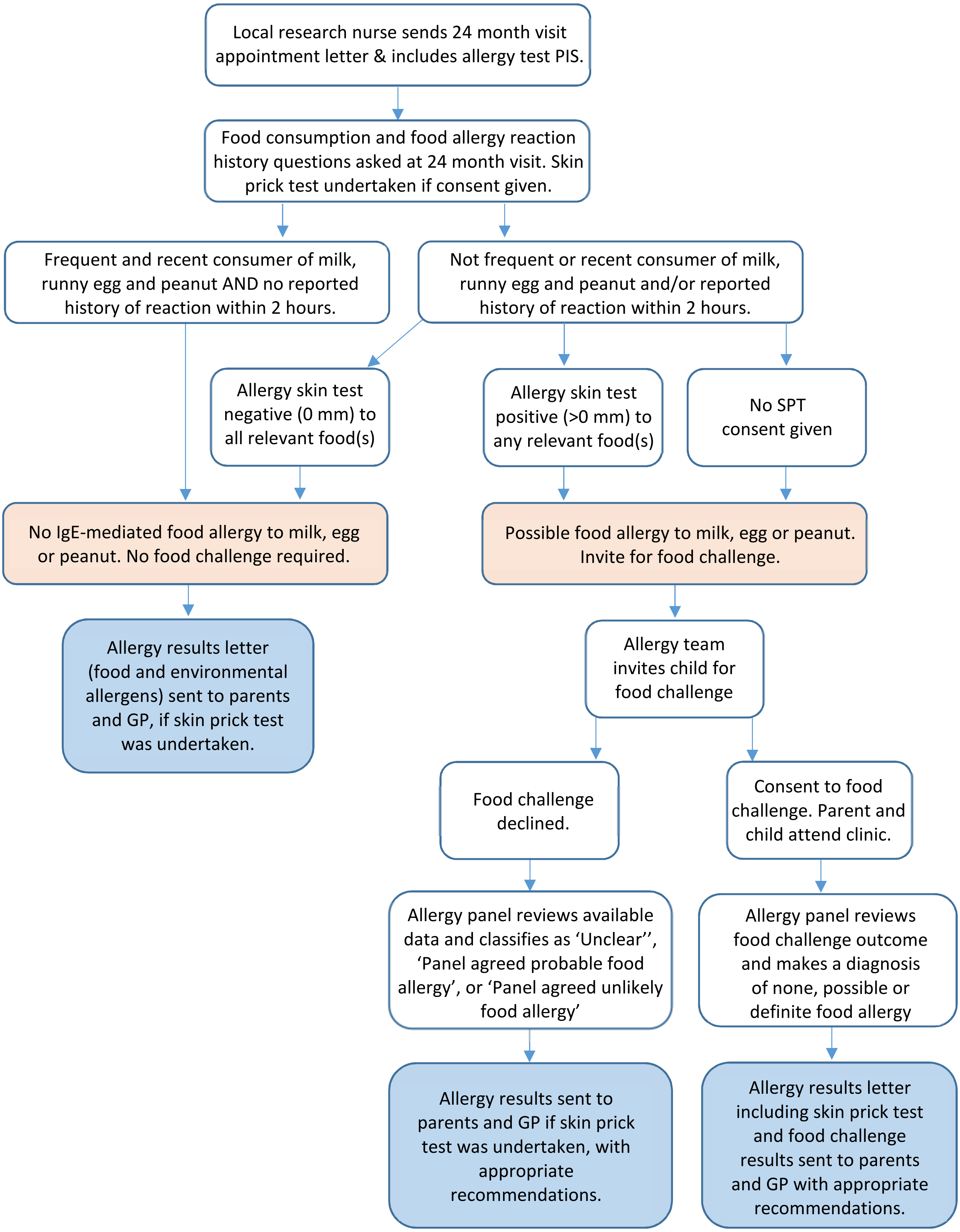

Figure S2: Detailed algorithm used to classify food allergy status for participants invited to oral food challenges but did not attend

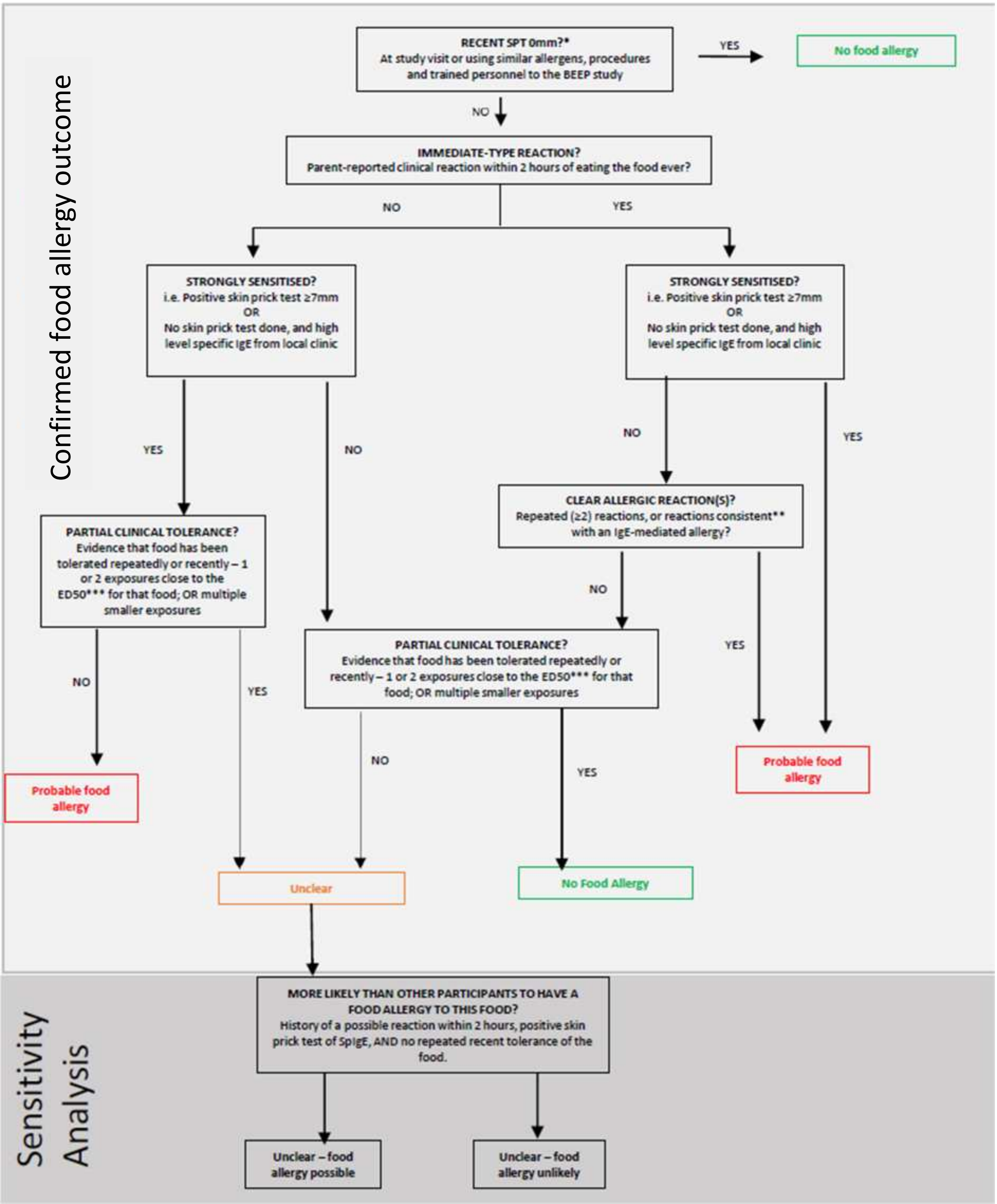

* Recent' is defined as within the year prior to the 2 year visit for milk or egg, and within 3 months prior to the 2 year visit for peanut.

Symptoms include urticaria, angioedema, vomiting/diarrhoea, sneezing, coughing, wheezing, stridor. Age at reaction and natural history of relevant food allergy are taken into consideration when assessing whether reaction is consistent with ongoing IgE-mediated allergy at age 2. * ED50 = eliciting dose at which 50% of people allergic to that food will react, estimated as 0.2g protein i.e. 5mls milk, 1.5g egg white, 0.7g peanut butter.

Changes to protocol after start of trial

Table S1: Changes to protocol after start of trial

Protocol	Date	Summary of changes
V2.1	15 Oct 2014	Clarification on inclusion and exclusion.
V2.2	13 Nov 2014	Updates include eligibility, both inclusion and exclusion criteria, details about the Skin Care Video, information to be collected 2 weeks post randomisation, questionnaire collection and timing.
V3.0	12 Oct 2015	Recruiting sites and PICs send text message to potential participants informing them of the trial and where to get further information.<br>At the 2 week follow up, the coordinating centre would like to text participants that have not responded to phone calls and/or emails.<br>Addition to the inclusion criteria which states that mothers must be aged ≥16 years.
V4.0	20 May 2016	Addition of food allergy outcome and tests, including Skin Prick Test at 24 month visit and the option of a Food Challenge after 24 month visit
V5.0	26 Oct 2016	Sample size revision proposed by TSC, Protocol and Information sheet updates to clarify procedures and safety around food allergy testing, and an additional letter for nurses to inform GP's that the child was seen for a 24 month visit.
V6.0	02 Aug 2017	Documents amended to attempt to increase retention rate at 24 months visit
V6.1	08 Nov 2017	Update to the BEEP Trial Skin Prick Testing Working Practice Document and clarification and added completion instructions on 36, 48 and 60 month questionnaires.

Skin care advice

Intervention group

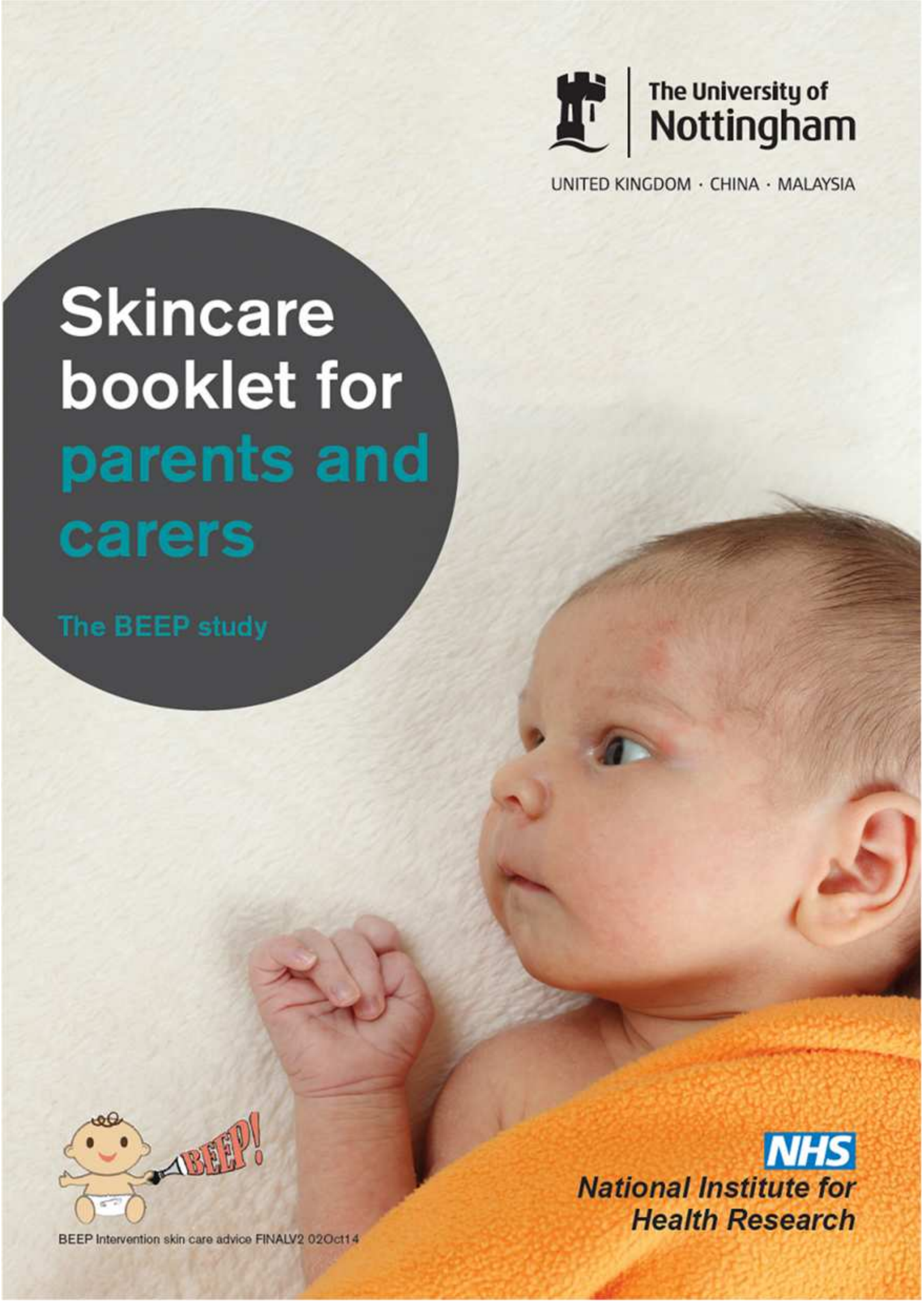

Welcome to

the BEEP study

We would like to take this opportunity to welcome you to the BEEP study and to thank you for taking part in this important NHS-funded research to look at preventing eczema.

This booklet will give you information on how to follow best practice skin care advice. Please take the time to read it and keep it safe so you can refer back to it during your time in the study.

We would like you to follow the recommendations in this booklet wherever you can.

If you would like to know more about the research that we do at the Centre of Evidence Based Dermatology, please visit **www.nottingham.ac.uk/dermatology**.

Thank you once again for helping with our study and for your important contribution to eczema research.

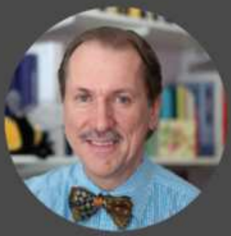

Professor Hywel C. Williams

Professor of Dermato-epidemiology and Consultant Dermatologist

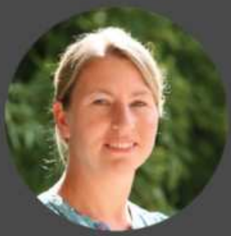

Dr Joanne Chalmers

Senior Research Fellow, Centre of Evidence based Dermatology

BEEP Intervention skin care advice FINALV2 02Oct14

Choosing which moisturiser to use

Moisturisers have two roles; they moisturise the skin and act as a barrier to protect the skin from irritants such as dribble and dirt. Choosing which moisturiser to use is down to personal preference. Different types of skin accept different moisturisers and some parents have a preference for a particular moisturiser. The moisturiser will be supplied to you free of charge for the first year that you and your baby are in the study.

In this study you can chose to use either **Diprobase cream** or **Doublebase gel.**

We will send you a sample of both moisturisers so you can decide which one you prefer.

During your time on the study, please avoid using any other moisturisers on your baby other than the one you have chosen. If you find you don't like the moisturiser you have chosen or it doesn't suit your baby's skin, you can change to the other whenever you wish by placing an order for the other moisturiser.

Applying the moisturiser

How? To apply the moisturiser, dot over the skin. Then smooth in using gentle downward strokes in the direction of the hair to coat the skin with a smooth film. Don't rub up and down the skin as this can block hair follicles and may cause a mild inflammation or infection of the hair follicles.

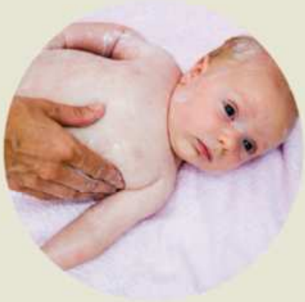

When? You should start applying the moisturiser as soon as possible after your baby is born. The latest should be three weeks after birth.

You should apply the moisturiser **at least once a day** (you can apply more often if you wish). It might help you remember if you find a regular time to do this, ie part of your daily routine. If your baby has a bath every day, then after the bath is ideal. Otherwise, after a nappy change, perhaps the last one before bedtime, is a good idea as your baby is already partly undressed. The changing mat, with a towel or sheet on top, is a good place to apply the moisturiser. **Always apply moisturiser after your baby has a bath** (even if you have already applied it that day) to compensate for the drying effects of the bath on your baby's skin.

You should apply the moisturiser all **over your baby** but it is OK to avoid the scalp as it will leave your baby's hair greasy. If your child is starting to stand/toddle we recommend you do not apply the moisturiser to the soles of their feet or wipe off any excess.

Remember your baby's skin will be slippery after you have applied the moisturiser, so take extra care when lifting your baby. Make sure you clean up any spillages on the floor and use a sheet or towel on the changing mat.

Don't worry if you forget to apply the moisturiser at least once a day, just apply it as soon as you have remembered.

BEEP Intervention skin care advice FINALV2 02Oct14

How do I order more moisturiser?

Once you have decided which moisturiser you want to use on your baby, please order more by sending an email to: **beep@nottingham.ac.uk**, stating your full name and the name of the moisturiser. We will then arrange for this to be posted to you.

When you have a quarter of a tub left, please send us another email to order more.

Suggestions for washing and bathing your baby

Soap and bubble bath can irritate and dry your baby's skin. Avoid using soap or adding bubble bath to your baby's bath. Instead, wash or bathe your baby using a mild cleanser which has been designed specially for babies. These products are less likely to irritate the skin and are less drying than soap.

Remember don't put your baby in a bath containing bubble bath which has been run for other children.

Bathing your baby two or three times a week is plenty, but you can bath your baby daily if you want to. Make sure the water isn't too hot.

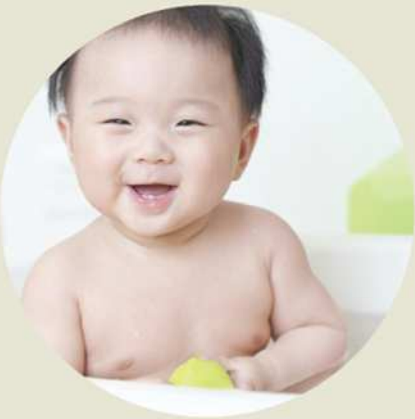

Moisturising bath oils and additives are not recommended for babies, unless they already have a skin problem. So avoid using these unless your doctor or nurse recommends it.

If you are using shampoo on your baby, make sure it is a mild shampoo designed specially for babies. When you rinse the shampoo, avoid washing the suds over the baby's body.

Try to avoid using wipes and lotions to clean your baby's hands and face as these areas are already exposed to more irritants during everyday life.

Changing your baby's nappy

Baby wipes are a convenient way of changing your baby's nappy, but they can irritate the skin, so it is best to avoid using them on your newborn baby if you can. The best way to change your baby's nappy is to use cotton wool and a bowl of warm water (with a mild cleanser if needed) instead, which will be less irritating to the skin.

Please remember these are general guidelines and you should follow the advice from your GP or health visitor for your own child.

BEEP Intervention skin care advice FINALV2 02Oct14

Top tips

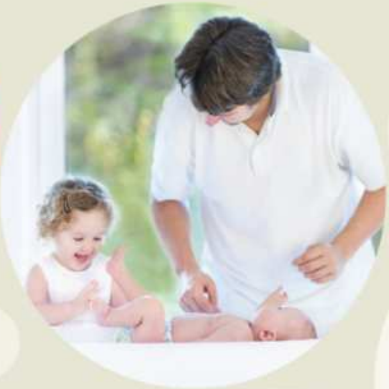

Try to find a regular time to apply the moisturiser so it becomes part of everyday life

When your baby has a bath, always apply the moisturiser afterwards, even if you have already applied it that day

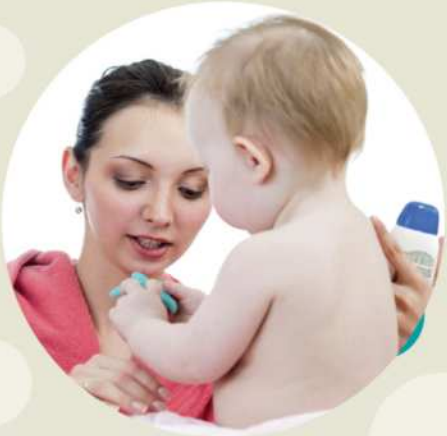

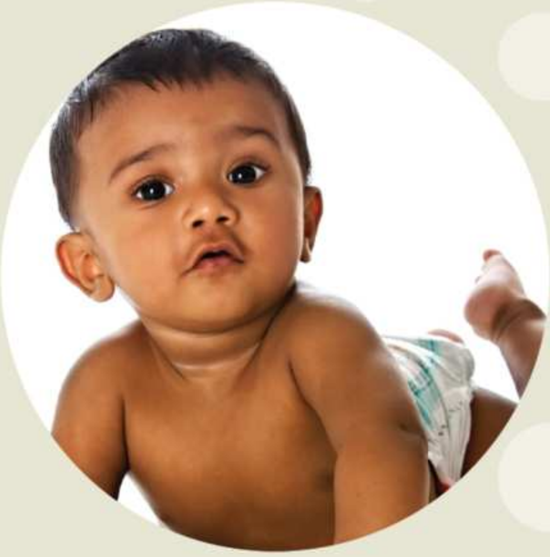

Remember not to use soap on your baby — instead, wash or bathe your baby using a mild cleanser designed specially for babies

BEEP Intervention skin care advice FINALV2 02Oct14

Frequently asked questions

What should I do if I spot a problem with my baby's skin? If your baby develops a rash or itchy skin you should contact your GP who can advise on the best action to take.

How do the moisturisers work? The moisturiser provides lipids (fats) to the skin to improve the barrier function of the skin. There have been previous studies in premature babies and shown to be safe and effective in keeping a healthy skin barrier.

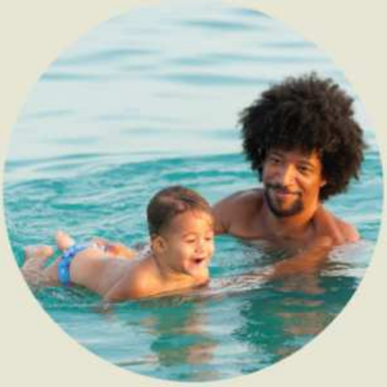

Can I take my baby swimming? Yes, but wash your baby afterwards and apply moisturiser.

What if my baby eats some of the moisturiser? The moisturiser should be kept out of reach of children, but any small amounts ingested, won't cause any harm.

Should I apply sun-cream before or after the moisturiser? If you want to put the moisturiser on in the daytime, put the moisturiser on first, then the sun-cream about half an hour later. Or you could just apply the moisturiser before bedtime, when you are not applying sun-cream.

What is meant by a defective skin barrier?

Healthy skin is like a brick wall (see picture). The bricks are the skin cells and the mortar is the fat (lipid) in between the bricks. The yellow fat acts like glue to stick the bricks together. The fat also helps keep water inside the skin cells. This makes the skin cells swell up, thereby closing any gaps between them. Healthy skin is like a strong brick wall that prevents allergens from the outside penetrating through the skin into the body.

In **eczematous skin**, there is not enough fat (mortar) in between the skin cells. As a result, water is lost from the skin cells and they shrink, opening up cracks in between them. The skin barrier is therefore abnormal in eczema. This allows penetration of allergens through the defective skin barrier. The skin reacts to the allergens, producing chemicals that trigger inflammation.

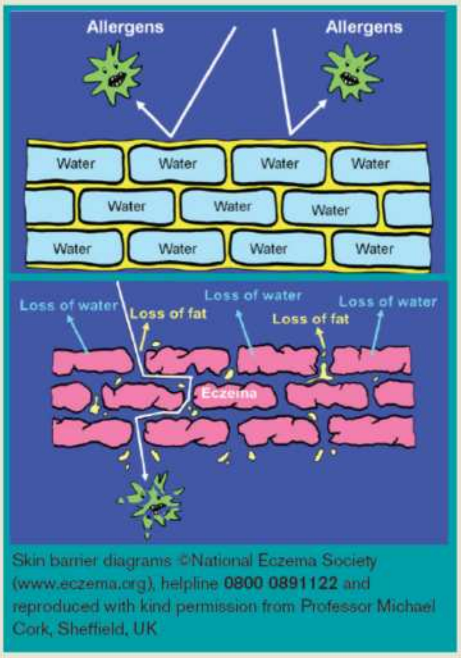

Skin barrier diagrams ©National Eczema Society (www.eczema.org), helpline **0800 0891122** and reproduced with kind permission from Professor Michael Cork, Sheffield, UK

BEEP Intervention skin care advice FINALV2 02Oct14

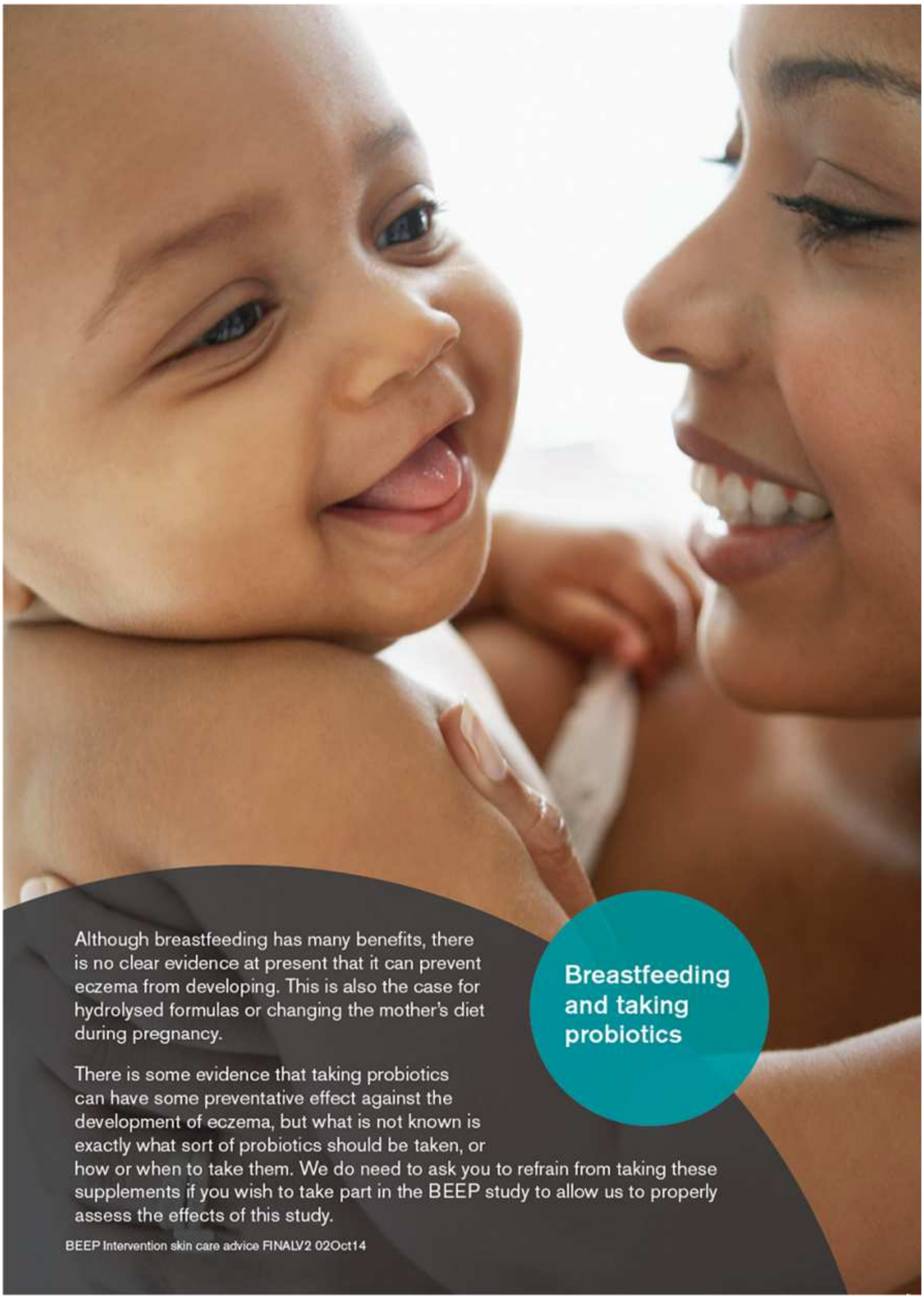

Breastfeeding and taking probiotics

Although breastfeeding has many benefits, there is no clear evidence at present that it can prevent eczema from developing. This is also the case for hydrolysed formulas or changing the mother's diet during pregnancy.

There is some evidence that taking probiotics can have some preventative effect against the development of eczema, but what is not known is exactly what sort of probiotics should be taken, or how or when to take them. We do need to ask you to refrain from taking these supplements if you wish to take part in the BEEP study to allow us to properly assess the effects of this study.

BEEP Intervention skin care advice FINALV2 02Oct14

Contact us

The BEEP study
Nottingham Clinical Trials Unit
Nottingham Health Science Partners
C Floor, South Block
Queens Medical Centre
Nottingham. NG7 2UH

t: 0115 884 4937
e: beep@nottingham.ac.uk
w: www.beepstudy.org

BEEP Intervention skin care advice FINALV2 02Oct14

Control group

Welcome to the BEEP study

We would like to take this opportunity to welcome you to the BEEP study and to thank you for taking part in this important NHS-funded research to look at preventing eczema.

This booklet will give you information on how to follow best practice skin care advice. Please take the time to read it and keep it safe so you can refer back to it during your time in the study.

We would like you to follow the recommendations in this booklet wherever you can.

If you would like to know more about the research that we do at the Centre of Evidence Based Dermatology, please visit **www.nottingham.ac.uk/dermatology**.

Thank you once again for helping with our study and for your important contribution to eczema research.

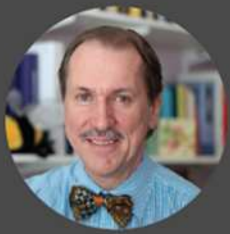

Professor Hywel C. Williams

Professor of Dermato-epidemiology and Consultant Dermatologist

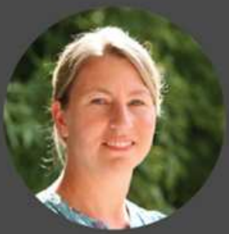

Dr Joanne Chalmers

Senior Research Fellow, Centre of Evidence based Dermatology

BEEP skin care advice FINAL V2 02Oct14

Suggestions for washing and bathing your baby

Soap and bubble bath can irritate and dry your baby's skin. Avoid using soap or adding bubble bath to your baby's bath. Instead, wash or bathe your baby using a mild cleanser which has been designed specially for babies. These products are less likely to irritate the skin and are less drying than soap.

Remember don't put your baby in a bath containing bubble bath which has been run for other children.

Bathing your baby two or three times a week is plenty, but you can bath your baby daily if you want to. Make sure the water isn't too hot.

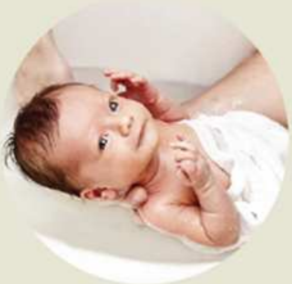

Moisturising bath oils and additives are not recommended for babies, unless they already have a skin problem. So avoid using these unless your doctor or nurse recommends it.

If you are using shampoo on your baby, make sure it is a mild shampoo designed specially for babies. When you rinse the shampoo, avoid washing the suds over the baby's body.Try to avoid using wipes and lotions to clean your baby's hands and face as these areas are already exposed to more irritants during everyday life.

Changing your baby's nappy

Baby wipes are a convenient way of changing your baby's nappy, but they can irritate the skin, so it is best to avoid using them on your newborn baby if you can. The best way to change your baby's nappy is to use cotton wool and a bowl of warm water (with a mild cleanser if needed) instead, which will be less irritating to the skin.

Breastfeeding and taking probiotics

Although breastfeeding has many benefits, there is no clear evidence at present that it can prevent eczema from developing. This is also the case for hydrolysed formulas or changing the mother's diet during pregnancy.

There is some evidence that taking some probiotics can have a preventative effect against the development of eczema, but what is not known is exactly what sort of probiotics should be taken, or how or when to take them. We do need to ask you to refrain from taking these supplements if you wish to take part in the BEEP study to allow us to properly assess the effects of this study.

Please remember these are general guidelines and you should follow the advice from your GP or health visitor for your own child.

BEEP skin care advice FINAL V2 02Oct14

Frequently asked questions

What should I do if I spot a problem with my baby's skin?

If your baby develops a rash or itchy skin you should contact your GP who can advise on the best action to take.

What is meant by a defective skin barrier?

Healthy skin is like a brick wall (see picture). The bricks are the skin cells and the mortar is the fat (lipid) in between the bricks. The yellow fat acts like glue to stick the bricks together. The fat also helps keep water inside the skin cells. This makes the skin cells swell up, thereby closing any gaps between them. Healthy skin is like a strong brick wall that prevents allergens from the outside penetrating through the skin into the body.

In **eczematous skin**, there is not enough fat (mortar) in between the skin cells. As a result, water is lost from the skin cells and they shrink, opening up cracks in between them. The skin barrier is therefore abnormal in eczema. This allows penetration of allergens through the defective skin barrier. The skin reacts to the allergens, producing chemicals that trigger inflammation.

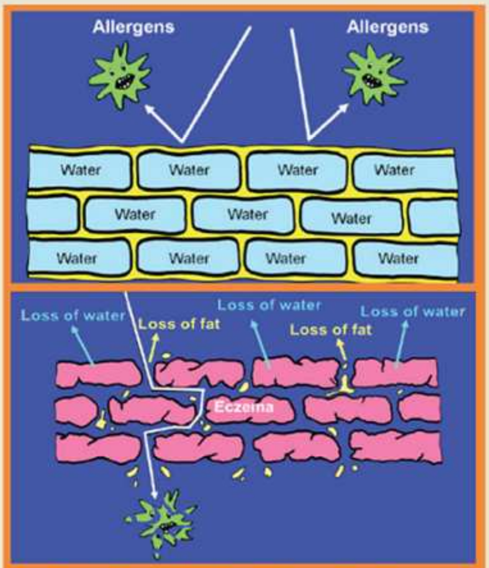

Skin barrier diagrams ©National Eczema Society (www.eczema.org), helpline **0800 0891122** and reproduced with kind permission from Professor Michael Cork, Sheffield, UK

Contact us

The BEEP study
Nottingham Clinical Trials Unit
Nottingham Health Science Partners
C Floor, South Block
Queens Medical Centre
Nottingham. NG7 2UH

t: 0115 884 4937
e: beep@nottingham.ac.uk
w: www.beepstudy.org

BEEP skin care advice FINAL V2 02Oct14

Study quality summaries

Table S2: Randomisation by recruiting centre

Site	Intervention (n = 693)	Control (n = 701)	Total (n = 1394)
Nottingham	95 (14%)	94 (13%)	189 (14%)
Portsmouth	47 (7%)	48 (7%)	95 (7%)
Harrogate	34 (5%)	34 (5%)	68 (5%)
King's Mill	43 (6%)	46 (7%)	89 (6%)
Burton	93 (13%)	95 (14%)	188 (13%)
Derby	54 (8%)	54 (8%)	108 (8%)
Leicester	36 (5%)	36 (5%)	72 (5%)
York	19 (3%)	19 (3%)	38 (3%)
Sheffield	103 (15%)	102 (15%)	205 (15%)
Imperial College	97 (14%)	97 (14%)	194 (14%)
Francis Grove Practice[1]	3 (<0.5%)	4 (1%)	7 (1%)
Streatham Common Group Practice[1]	1 (<0.5%)	1 (<0.5%)	2 (<0.5%)
Clapham Park Group Practice[1]	2 (<0.5%)	3 (<0.5%)	5 (<0.5%)
Park Group Practice[1]	1 (<0.5%)	2 (<0.5%)	3 (<0.4%)
Guy's & St Thomas	38 (5%)	39 (6%)	77 (6%)
Bristol	27 (4%)	27 (4%)	54 (4%)

[1] *GP practices are treated as one recruiting centre in the analysis.*

Table S3: Questionnaire completion rates at 3, 6, 12 and 18 months

	Intervention (n = 693)	Control (n = 701)	Total (n = 1394)
**3 months**	534 (77%)	524 (75%)	1058 (76%)
**6 months**	530 (76%)	521 (74%)	1051 (75%)
**12 months**	523 (75%)	535 (76%)	1058 (76%)
**18 months**	497 (72%)	512 (73%)	1009 (72%)

Table S4: 2 year visit completion rate

	Intervention (n = 693)	Control (n = 701)	Total (n = 1394)
**2 year visit completed**	599 (86%)	613 (87%)	1212 (87%)
**Type of visit**			
Face to face	555 (80%)	568 (81%)	1123 (81%)
Telephone/Email/SMS	32 (5%)	35 (5%)	67 (5%)
Post	12 (2%)	10 (1%)	22 (2%)

Table S5: Genetic study (*FLG)* completion rate

	Intervention (n = 693)	Control (n = 701)	Total (n = 1394)
Consented to genetic study (of those completing the 2-year visit)			
no	40 (6%)	34 (5%)	74 (5%)
yes	559 (81%)	579 (83%)	1138 (82%)
Saliva sample for DNA extraction collected at visit			
no	103 (15%)	96 (14%)	199 (14%)
yes	456 (66%)	483 (69%)	939 (67%)
Sample tested	485 (70%)	506 (72%)	991 (71%)
Result obtained on 4 *FLG* mutations tested (all children)	477 (69%)	491 (70%)	968 (69%)
**Included in analysis of *FLG genotype***	402 (58%)	414 (59%)	816 (59%)
Both parents of white ethnicity	*400 (58%)*	*410 (58%)*	*810 (58%)*
Mutation detected	*2 (<0.5%)*	*4 (1%)*	*6 (<0.5%)*
**Not included in *FLG* analysis**	291 (42%)	287 (41%)	578 (41%)
No mutation detected – unable to exclude a mutation due to ethnicity.	75 (11%)	80 (11%)	155 (11%)
Genotyping failed	8 (1%)	12 (2%)	20 (1%)
No sample[1]	74 (11%)	72 (10%)	146 (10%)
No consent	40 (6%)	34 (5%)	74 (5%)
No 24 month visit	94 (14%)	88 (13%)	182 (13%)
Other[2]	-	1 (<0.5%)	1 (<0.5%)

1 – *visit not done face to face or sample not collected at visit and kit not left, or the parents did not return sample.*
2 – *Sample collected at visit but no record of being sent for testing.*

Table S6: Skin prick test completion rate

	Intervention	Control	Total
**Total number with 2 year visit data**	599	613	1212
Consent to skin prick test			
no	49 (8%)	58 (9%)	107 (9%)
yes	508 (85%)	512 (84%)	1020 (84%)
n/a - not face to face	42 (7%)	43 (7%)	85 (7%)
Skin prick test completion			
SPT not done[1]	11 (2%)	7 (1%)	18 (1%)
SPT done	484 (81%)	494 (81%)	978 (81%)
SPT partially done	13 (2%)	11 (2%)	24 (2%)
Adverse reactions to SPT[2]			
Systemic reaction reported[3]	-	1	1
Antihistamine given	4	1	5
Adrenaline auto-injector used	-	-	-

Percentages use the total number of follow-up visits completed as the denominator.

1 – *Reasons for SPT not being done: child unwell (n=1), child not willing or distressed (n=12), did not consent to all allergens being tested (n=1), parent withdrew consent (n=3), not enough lancets (n=1).*

2 – *information not completed for one participant where SPT completed.*

3 – *Additional information: "Slight cough- was eating chocolate. Seen by doctor in case it was a reaction to the skin prick test. Documented in the medical notes."*

Table S7: Food allergy assessment rate

	Intervention	Control	Total
**Total number of follow-up visits completed**	599	612	1212
**Food challenge required**			
For peanut	76 (13%)	85 (14%)	161 (13%)
For cow's milk	32 (5%)	28 (5%)	60 (5%)
For egg	83 (14%)	73 (12%)	156 (13%)
For at least one of these three foods	133 (22%)	125 (20%)	258 (21%)
**Food challenge took place**[1]			
For peanut	22 (4%)	19 (3%)	41 (3%)
For cow's milk	5 (1%)	1 (<0.5%)	6 (<0.5%)
For egg	24 (4%)	11 (2%)	35 (3%)
For at least one of these three foods	41 (7%)	28 (5%)	69 (6%)
**Diagnosis decision where food challenge required**			
For peanut			
by food challenge	22 (29%)	19 (22%)	41 (25%)
panel diagnosis – probable food allergy/no food allergy[2]	33 (43%)	40 (47%)	73 (45%)
panel diagnosis – possible food allergy/food allergy unlikely unclear[3]	21 (28%)	26 (31%)	47 (29%)
n	76	85	161
For cow's milk			
by food challenge	5 (16%)	1 (4%)	6 (10%)
panel diagnosis – probable food allergy/no food allergy[2]	22 (69%)	22 (79%)	44 (73%)
panel diagnosis – possible food allergy/food allergy unlikely unclear[3]	5 (16%)	5 (18%)	10 (17%)
n	32	28	60
For egg			
by food challenge	24 (29%)	11 (15%)	35 (22%)
panel diagnosis – probable food allergy/no food allergy[2]	43 (52%)	45 (62%)	88 (56%)
panel diagnosis – possible food allergy/food allergy unlikely unclear[3]	16 (19%)	17 (23%)	33 (21%)
n	83	73	156

Children could have food challenges for more than one food.
1 – *Blinding was maintained for all oral food challenges*
2 – *Panel consensus decision of probable food allergy or no food allergy included in main analysis of confirmed food allergy outcome at 24 months.*
3 – *Panel consensus decision of food allergy possible or food allergy unlikely included in sensitivity analysis for the main food allergy outcome at 24 months.*

Table S8: Adherence; parent report of use of study emollient in the intervention group during the first year

Parental report of first emollient use	Intervention (n = 693)
Days between birth and parental report of first emollient use	
Mean [sd]	12.8 [9.6]
Median [25th, 75th centile]	11 [7, 17]
Min, max	1, 104
n	509
Not known	184 (27%)
Age when started using emollient	
Three days old or less	43 (8%)
Between 4 and 7 days old	115 (23%)
Between 8 and 14 days old	187 (37%)
Between 15 and 21 days old	107 (21%)
3 to 6 weeks old	51 (10%)
Older than 6 weeks	6 (1%)
n	509

Percentage for age when started using emollient uses the number of children with data as the denominator.

3 months	Intervention
Usual frequency of emollient use	
Never	20 (4%)
Once or twice a week	34 (6%)
3 or 4 days a week	75 (14%)
5 or 6 days a week	104 (20%)
Everyday	299 (56%)
n	532
Emollient usually applied to:	
Face/neck	398 (75%)
Arms/legs	508 (95%)
Trunk	483 (91%)
At least two of the areas above	495 (93%)
n	533
Usual number of applications per day	
None	17 (3%)
Once	422 (79%)
Twice	63 (12%)
More than twice	30 (6%)
n	532
Emollient use after bathing/showering child	
Never	21 (4%)
Sometimes	40 (8%)
Usually	471 (89%)
n	532
Reasons if emollient not used at all in past 3 months	
Prescribed or advised to use a different emollient	3
Advised to stop applying emollient altogether	1
Not enough time to apply emollient	4
Baby didn't like	-
Ran out of emollient	-
Other	12
n	20
Used emollient at least three days per week over the majority of the child's body during the past 3 months	
No	66 (12%)
Yes	466 (88%)
n	532

6 months	Intervention
Usual frequency of emollient use	
Never	33 (6%)
Once or twice a week	53 (10%)
3 or 4 days a week	61 (12%)
5 or 6 days a week	84 (16%)
Everyday	289 (56%)
n	520
Emollient usually applied to:	
Face/neck	397 (76%)
Arms/legs	480 (92%)
Trunk	464 (89%)
At least two of the areas above	472 (91%)
n	519
Usual number of applications per day	
None	31 (6%)
Once	382 (74%)
Twice	68 (13%)
More than twice	36 (7%)
n	517
Emollient use after bathing/showering child	
Never	37 (7%)
Sometimes	38 (7%)
Usually	441 (85%)
n	516
Reasons if emollient not used at all in past 3 months	
Prescribed or advised to use a different emollient	18
Advised to stop applying emollient altogether	-
Not enough time to apply emollient	2
Baby didn't like	1
Ran out of emollient	-
Other	10
n	31
Used emollient at least three days per week over the majority of the child's body during the past 3 months	
No	92 (18%)
Yes	427 (82%)
n	519

12 months	Intervention
Usual frequency of emollient use	
Never	58 (11%)
Once or twice a week	68 (13%)
3 or 4 days a week	74 (15%)
5 or 6 days a week	59 (12%)
Everyday	248 (49%)
n	507
Emollient usually applied to:	
Face/neck	351 (69%)
Arms/legs	448 (88%)
Trunk	427 (84%)
At least two of the areas above	438 (86%)
n	508
Usual number of applications per day	
None	50 (10%)
Once	362 (72%)
Twice	69 (14%)
More than twice	25 (5%)
n	506
Emollient use after bathing/showering child	
Never	58 (11%)
Sometimes	44 (9%)
Usually	406 (80%)

12 months	Intervention
n	508
Reasons if emollient not used at all in past 6 months	
Prescribed or advised to use a different emollient	31
Advised to stop applying emollient altogether	2
Not enough time to apply emollient	4
Baby didn’t like	4
Ran out of emollient	2
Other	14
n	57
Used emollient at least three days per week over the majority of the child’s body during the past 3 months	
No	131 (26%)
Yes	375 (74%)
n	506

Table S9: Contamination; parent report of (non-study) self-directed emollient use in the control group during the first year

All children	Control
**3 months**	
Applied moisturiser to baby's skin or used oil for baby massage at least three days per week over most or all of the child's body during the past 3 months	
No	407 (79%)
Yes	110 (21%)
n	517
**6 months**	
Applied moisturiser to baby's skin or used oil for baby massage at least three days per week over most or all of the child's body during the past 3 months	
No	370 (72%)
Yes	143 (28%)
n	513
**12 months**	
Applied moisturiser to baby's skin or used oil for baby massage at least three days per week over most or all of the child's body during the past 6 months	
No	376 (72%)
Yes	144 (28%)
n	520

Excluding children with eczema (i.e. those that may have been using emollients for treating eczema)	
**3 month questionnaire and no reported eczema**	n = 457
Applied moisturiser to baby's skin or used oil for baby massage at least three days per week over most or all of the child's body during the past 3 months	
No	375 (82%)
Yes	82 (18%)
**6 month questionnaire and no reported eczema at 3 or 6 months**	n = 372
Applied moisturiser to baby's skin or used oil for baby massage at least three days per week over most or all of the child's body during the past 3 months	
No	310 (83%)
Yes	62 (17%)
**12 month questionnaire and no reported eczema at 3, 6 or 12 months**	n = 324
Applied moisturiser to baby's skin or used oil for baby massage at least three days per week over most or all of the child's body during the past 6 months	
No	275 (85%)
Yes	49 (15%)

Questionnaires asked about applying moisturisers to the baby's skin or used oil for baby massage

Report of eczema on a questionnaire is defined as a response of "yes" to "In the last xx months, has your baby been diagnosed with eczema by a doctor or a nurse?"

Table S10: Summary of adherence and contamination during the first year

Children with complete data by allocated group	Intervention (n = 442)	Control (n = 439)
Level of compliance in the intervention group/contamination in the control group		
Full	311 (70%)	56 (13%)
Early onset application	86 (19%)	38 (9%)
Late onset application	17 (4%)	85 (19%)
None	28 (6%)	260 (59%)

Early onset application is during the first 3 months, late onset application is application at 6 and/or 12 months (but not at 3 months).

For all children by allocated group	Intervention (n = 693)	Control (n = 701)
Level of compliance in the intervention group/contamination in the control group		
Full	350 (51%)	74 (11%)
Early onset application	153 (22%)	59 (8%)
Late onset application	21 (3%)	90 (13%)
None	169 (24%)	478 (68%)

See methods sections above for details on assumptions used for children with incomplete data on compliance/contamination

Table S11: List of Protocol deviations (as reported by research nurses on the eCRF) not relating to food allergy assessment process

Participant number	Group	Days to deviation from randomisation	Deviation	Further details on deviation (as entered by nurse on eCRF)
1007	Usual care	0	inclusion/exclusion criteria deviation	Baby was older than 21 days
1061	Intervention	0	treatment randomisation error	one adult only with atopic disease not three
2057	Usual care	0	treatment randomisation error	Atopic catagory on macro is correct 3
2062	Intervention	0	treatment randomisation error	Atopic catagory on randomisation should be 2
4074	Intervention	0	inclusion/exclusion criteria deviation	DOB given at baseline was incorrect,
5190	Intervention	0	treatment randomisation error	1st degree relative with atopic disease 1 not 2
6021	Intervention	0	treatment randomisation error	Randomised in error
6039	Usual care	0	treatment randomisation error	CTU incorrect 1st degree relative should be 2
6104	Usual care	0	treatment randomisation error	number of family with atopic disease has changed
7001	Intervention	0	treatment randomisation error	incorrect number of Atopic category entered
7005	Usual care	0	treatment randomisation error	incorrect Atopic category number entered
7006	Usual care	-1	treatment randomisation error	Randomisation error ,incorrect value entered .
8001	Usual care	0	trial procedure not performed per protocol	randomised out of time window
8012	Intervention	-29	informed consent deviation	No consent form could be found in the site file 27
9199	Usual care	0	treatment randomisation error	2 atopic family members recorded, should be 3
10039	Usual care	0	inclusion/exclusion criteria deviation	Trial manager permits randomisation at 22 days
10209	Usual care	0	treatment randomisation error	2 relatives with hx, recorded in error as 1
10216	Intervention	0	treatment randomisation error	2 relatives with hx recorded in error as 3

Table S12: List of Protocol deviations (as reported by research nurses on the eCRF) relating to food allergy assessment process

Protocol deviations related to skin prick tests

	Intervention	Control
Full fat milk used	96	94
Semi skimmed milk used	3	5
Incorrect milk used (type not specified)	-	1
Incorrect cat & dust mite allergen used	7	7
Incorrect dust mite allergen used	4	7

Protocol deviations related to oral food challenge

Participant number	Group	Days to deviation from randomisation	Deviation	Further details on deviation (as entered by nurse on eCRF)
3037	Intervention	794	participant non-compliance with protocol	patient declined to eat dose 4& 5 of peanut butter
10081	Intervention	918	trial procedure not performed per protocol	Dr XXXX advised to skip first 2 doses
10144	Intervention	783	other	Food challanged last dose given on different day
15006	Intervention	819	trial procedure not performed per protocol	Dr XXXX advised to skip 4th Dose as child refused
15009	Intervention	902	trial procedure not performed per protocol	Dr XXXX advised to skip 1st dose

Results

Table S13: Frequency of bathing/showering of children

6 month questionnaire	Intervention	Control
**Bath/shower frequency**		
Less than once a week	14 (3%)	9 (2%)
Approx. once a week	86 (17%)	104 (20%)
Approx. every other day	210 (41%)	191 (37%)
Every day or most days	207 (40%)	207 (41%)
n	517	511
**Products used to wash child**		
Water only	168 (32%)	156 (31%)
Wash product only	258 (50%)	256 (51%)
Emollient only	35 (7%)	39 (8%)
Something else only	17 (3%)	31 (6%)
Wash product & emollient	18 (3%)	12 (2%)
Wash product & something else	15 (3%)	9 (2%)
Emollient & something else	2 (<0.5%)	1 (<0.5%)
Other	4 (1%)	2 (<0.5%)
n	517	506
**Regularly used oils in the child's bath water**		
No	475 (92%)	452 (89%)
Yes	41 (8%)	54 (11%)
n	516	506

12 month questionnaire	Intervention	Control
**Bath/shower frequency**		
Less than once a week	4 (1%)	7 (1%)
Approx. once a week	64 (13%)	59 (11%)
Approx. every other day	181 (36%)	201 (38%)
Every day or most days	260 (51%)	256 (49%)
n	509	523
**Products used to wash child**		
Water only	148 (29%)	123 (24%)
Wash product only	266 (52%)	303 (58%)
Emollient only	39 (8%)	40 (8%)
Something else only	27 (5%)	22 (4%)
Wash product & emollient	10 (2%)	15 (3%)
Wash product & something else	11 (2%)	16 (3%)
Emollient & something else	3 (1%)	2 (<0.5%)
Other	3 (1%)	2 (<0.5%)
n	507	523
**Regularly used oils in the child's bath water**		
No	446 (89%)	456 (88%)
Yes	57 (11%)	60 (12%)
n	503	516

24 month questionnaire	Intervention	Control
**Bath/shower frequency**		
Less than once a week	3 (1%)	2 (<0.5%)
Approx. once a week	53 (9%)	52 (9%)
Approx. every other day	244 (43%)	256 (43%)
Every day or most days	271 (47%)	286 (48%)
n	571	596
**Products used to wash child**		
Water only	83 (15%)	97 (16%)
Wash product only	381 (67%)	374 (63%)
Emollient only	43 (8%)	42 (7%)
Something else only	15 (3%)	26 (4%)

24 month questionnaire	Intervention	Control
Wash product & emollient	24 (4%)	32 (5%)
Wash product & something else	13 (2%)	18 (3%)
Emollient & something else	4 (1%)	3 (1%)
Other	6 (1%)	2 (<0.5%)
n	569	594
**Regularly used oils in the child's bath water**		
No	528 (92%)	540 (91%)
Yes	44 (8%)	56 (9%)
n	572	596

Table S14: Other post-randomisation practices

6 month questionnaire	Intervention (n = 530)	Control (n = 521)
**How baby was fed between birth and 6 months**		
Breast milk only	179 (35%)	190 (37%)
Formula milk only	90 (17%)	75 (15%)
Mixed breast and formula feeding	248 (48%)	245 (48%)
Other	1 (<0.5%)	-
n	518	510
**Solid food introduced by 6 months**	476 (92%)	484 (95%)
n	517	508
**Mother had antibiotics whilst breastfeeding[1]**	110 (26%)	129 (30%)
Could not remember	1 (<0.5%)	2 (<0.5%)
n	427	435
**Number of courses of antibiotics**		
Median [25th, 75th centile]	1 [1, 2]	1 [1, 2]
Min, max	1, 7	1, 40
n	105	127
Didn't know/didn't complete	5	2
**Mother regularly took probiotics whilst breastfeeding[1]**	28 (7%)	24 (6%)
n	423	431
**Baby given regular probiotic supplement between birth and 6 months**	20 (4%)	8 (2%)
n	516	507

1 – *tabulated for babies who were not fed using formula milk only between birth and 6 months.*

12 month questionnaire	Intervention (n = 523)	Control (n = 535)
**Baby had antibiotics between birth and 1 year**		
Yes	193 (38%)	212 (40%)
Could not remember	6 (1%)	1 (<0.5%)
n	508	524
**Number of courses of antibiotics**		
Median [25th, 75th centile]	1 [1, 2]	1 [1, 2]
Min, max[1]	0, 16	1, 90
n	184	201
Didn't know/didn't complete	9	11

1 – *One participant responded as "yes" to "Over the last year, has your baby had any antibiotics?", then responded as "0" to "If yes, how many courses?".*

24 month questionnaire	Intervention (n = 599)	Control (n = 613)
**Age in months when child first had solids**		
Median [25th, 75th centile]	6 [5, 6]	6 [5, 6]
Min, max	2, 12	3, 18
n	572	595
**Baby had antibiotics between 1 and 2 years**		
Yes	266 (47%)	281 (47%)
Could not remember	9 (2%)	12 (2%)
n	572	596
**Number of courses of antibiotics**		
Median [25th, 75th centile]	1 [1, 2]	1 [1, 2]
Min, max	1, 18	1, 12
n	265	278
Didn't know	1	3
**Baby had antibiotics between birth and 2 years**		
Yes	342 (59%)	347 (58%)
No/could not remember at 24,	46 (8%)	58 (10%)

24 month questionnaire	Intervention (n = 599)	Control (n = 613)
not information at 12		
n	578	600
**Additional children in house since baby born**	54 (9%)	57 (10%)
n	572	596
**Number of other children in household at 24 months**		
0	203 (36%)	225 (38%)
1	257 (45%)	249 (42%)
2	78 (14%)	83 (14%)
3 or more	33 (6%)	38 (6%)
n[1]	571	595
**Family has any furry pets that live entirely or partly in the house**		
Yes	217 (38%)	261 (44%)
n	572	596
Type of furry pet		
Dog	119 (21%)	134 (22%)
Cat	99 (17%)	139 (23%)
Other	35 (6%)	39 (7%)
**Furry pets introduced into house since birth**	25/572 (4%)	43/596 (7%)
**Attempted to reduce dust mites in 2 years since birth**	211 (37%)	178 (30%)
n	572	596
**Water softener fitted in house**	17 (3%)	21 (4%)
Yes		
n	572	594
**Child regularly attended nursery or playgroup**	428 (75%)	459 (77%)
n	572	596

1 – *for two infants, the parents indicated that there were more children in the household compared to when the baby was born but did not say how many.*

Table S15. Timing of introduction of allergenic foods asked at 24 months

	Intervention	Control
**Child ever eaten food containing cow's milk**		
No	6 (1%)	5 (1%)
Yes	569 (99%)	593 (99%)
n	575	598
Age in months the first time they had food containing cow's milk		
Median [25th, 75th centile]	6 [1, 7]	6 [1, 7]
Min, max	0, 22	0, 22
n	566	591
Before 4 months	189 (33%)	212 (36%)
Between 4 and 6 months	233 (41%)	224 (38%)
Between 7 and 12 months	129 (23%)	144 (24%)
Between 13 and 24 months	15 (3%)	11 (2%)
After 24 months/never eaten	6 (1%)	5 (1%)
n	572	596
**Child ever eaten food containing egg**		
No	10 (2%)	8 (1%)
Yes	565 (98%)	590 (99%)
n	575	598
Age in months the first time they had food containing egg		
Median [25th, 75th centile]	7 [6, 9]	7 [6, 9]
Min, max	4, 21	1, 24
n	562	587
Before 4 months	-	1 (<0.5%)
Between 4 and 6 months	251 (44%)	244 (41%)
Between 7 and 12 months	284 (50%)	319 (54%)
Between 13 and 24 months	27 (5%)	23 (4%)
After 24 months/never eaten	10 (2%)	8 (1%)
n	572	595
**Child ever eaten food containing peanut**		
No	138 (24%)	146 (24%)
Yes	436 (76%)	452 (76%)
n	574	598
Age in months the first time they had food containing peanut		
Median [25th, 75th centile]	12 [8, 14]	12 [8, 14]
Min, max	3, 24	3, 25
n	434	450
Before 4 months	1 (<0.5%)	1 (<0.5%)
Between 4 and 6 months	62 (11%)	63 (11%)
Between 7 and 12 months	245 (43%)	251 (42%)
Between 13 and 24 months	126 (22%)	134 (22%)
After 24 months/never eaten	138 (24%)	147 (25%)
n	572	596
**Child ever eaten food containing nuts other than peanut**		
No	109 (19%)	112 (19%)
Yes	465 (81%)	485 (81%)
n	574	597
Age in months the first time they had food containing nuts other than peanut		
Median [25th, 75th centile]	12 [10, 18]	12 [12, 18]
Min, max	4, 26	4, 24
n	462	484
Before 4 months	-	-
Between 4 and 6 months	36 (6%)	35 (6%)
Between 7 and 12 months	278 (49%)	261 (44%)
Between 13 and 24 months	147 (26%)	188 (32%)
After 24 months/never eaten	110 (19%)	112 (19%)
n	571	596

Table S16: Sensitivity analyses for primary outcome

a) Additional adjustment for variables with baseline imbalance

No important differences between groups in baseline variables were identified so this analysis was not done.

b) Using data from GP records for participants with no primary outcome data

	Intervention	Control	Adjusted relative risk (95% CI)	Adjusted difference in risk (95% CI)
Atopic eczema in the previous 12 months using the UK working party **diagnostic criteria** for under 4s	139/598 (23%)	150/612 (25%)		
Eczema between 12 and 24 months ascertained from **GP records** for participants with no primary outcome data	6/26 (23%)	7/27 (26%)		
Atopic eczema in the previous 12 months using the UK working party **diagnostic criteria** for under 4s **or** eczema ascertained from **GP records**	145/624 (23%)	157/639 (25%)	0.95 (0.78 to 1.15)	-1.2% (-5.9% to 3.5%)

Eczema between 12 and 24 months ascertained from GP records defined as diagnosis of eczema made between 12 or 24 months or a prescription for eczema between 18 and 24 months if the diagnosis of eczema was made before 12 months.
Adjusted relative risk/difference in risk estimated using the same model as for the primary outcome (see Table 2 in main paper)

c) According to method of data collection (face to face or by phone/email/text)

	Intervention (n = 598)	Control (n = 612)	Adjusted relative risk (95% CI)	Adjusted difference in risk (95% CI)
**Data collected at face to face visit**				
Atopic eczema in the previous 12 months using the UK working party diagnostic criteria for under 4s	131/555 (24%)	142/568 (25%)	0.95 (0.77 to 1.16)	-1.3% (-6.3% to 3.7%)
**Data collected by phone/email/text**				
Atopic eczema in the previous 12 months using the UK working party diagnostic criteria for under 4s	8/43 (19%)	8/44 (18%)	1.02 (0.43 to 2.40)	-1.4% (-17.7 to 14.9)

Adjusted relative risk/difference in risk estimated using the same model as for the primary outcome

d) Replacing the UK working party criteria definition of visible dermatitis with visible dermatitis **anywhere** on the body (using the EASI assessment)

	Intervention (n = 598)	Control (n = 612)	Adjusted relative risk (95% CI)	Adjusted difference in risk (95% CI)
Atopic eczema in previous 12 months	147 (25%)	161 (26%)	0.93 (0.77 to 1.14)	-1.5% (-6.4% to 3.4%)

Adjusted relative risk/difference in risk estimated using the same model as for the primary outcome
Note visible dermatitis anywhere on the body defined as an EASI score of more than 0 which was assessed during the face to face visit at 24 months (n = 1120) or any visible flexural dermatitis (n = 75) if the EASI assessment was not done. Note for 15 participants, information on visible dermatitis was not available: 13 with a response of "no" to "In the last year, has your child had an itchy skin condition -by itchy we mean scratching or rubbing the skin a lot?" and 2 with a response of "yes" to the question about itchy skin in the last year who also responded as "yes" to the question about the skin condition affecting the cheeks or skin creases and the question about generally dry skin.

e) Using imputation for missing outcome data

	Intervention (n = 693)	Control (n = 701)	Adjusted relative risk (95% CI)	Adjusted difference in risk (95% CI)
Using multiple imputation model as specified in SAP[1]	23.3% (SE 1.7%)	24.5% (SE 1.8%)	0.96 (0.78to 1.17)	-1.0% (-5.8% to 3.8%)
Multiple imputation model including randomisation stratification variables only[2]	23.0% (SE 1.7%)	24.6% (SE 1.7%)	0.94 (0.77 to 1.14)	-1.3% (-6.0% to 3.3%)
Assuming participants with missing outcome in the intervention group are eczema free and in the usual care group have atopic eczema (best case scenario)	139 (20%)	239 (34%)	0.60 (0.49 to 0.71)	-13.9% (-18.5% to -9.3%)
Assuming participants with missing outcome in the intervention group have atopic eczema and in the usual care group are eczema free (worst case scenario)	234 (34%)	150 (21%)	1.58 (1.32 to 1.88)	12.5% (7.9% to 17.1%)

1 – *Variables included in the multiple imputation are: UK working party diagnosis of eczema at 24 months, parental report of a clinical diagnosis of eczema at 24 months, confirmed diagnosis of food allergy to any of milk, egg or peanut at 24 months, parental report of immediate allergy to cow's milk, egg or peanut at 24 months, allergic sensitisation to cow's milk, egg or peanut, centre, number of immediate family members with atopic disease, number of FLG null mutations (none, one or two), the number of immediate family members with eczema, water hardness, season of birth and regular use of probiotic supplements during pregnancy, number of other children in the household at randomisation, furry pets at randomisation, summary variable for compliance/contamination in first year of life and allocated group. Analysis assumes that missing outcome data are missing at random and depend on the observed variables included in the imputation model but not the unobserved outcomes. Forty datasets were imputed and estimates were combined using Rubin's rules. Adjusted relative risk/difference in risk estimated using the same model as for the primary outcome*

2 - *The analysis was also repeated using simpler multiple imputation model including the outcomes of UK working party diagnosis of eczema at 24 months, confirmed diagnosis of food allergy to any of milk, egg or peanut at 24 months, allocated group and the randomisation stratification variables of centre, number of immediate family members with atopic disease with 20 imputations.*

Table S17: Complier average causal effect for primary outcome of eczema between the age of 1 and 2 years

a) Summary of compliance/contamination and eczema between the age of 1 and 2 years

	Eczema between the age of 1 and 2 years	
**Level of compliance/contamination**	Intervention n (%)	Control n (%)
Participants with complete data on compliance/contamination		
Full	67/308 (22%)	24/54 (44%)
Early onset application[1]	27/82 (33%)	11/37 (30%)
Late onset application[2]	3/15 (20%)	39/84 (46%)
None	4/26 (15%)	34/255 (13%)
All participants with primary outcome available		
Full	74/345 (21%)	32/71 (45%)
Early onset application[1]	38/135 (28%)	18/54 (33%)
Late onset application[2]	3/19 (16%)	40/89 (45%)
None	24/99 (24%)	60/398 (15%)

[1] *Early onset application is application during the first 3 months (with neither or only one of 6 or 12 months)*
[2] *Late onset application is application at 6 and/or 12 months, but not at 3 months.*

b) Complier average causal effect for eczema between the age of 1 and 2 years

	Adjusted odds ratio (95% CI)
Compliance defined as widespread emollient use over the child's body at least three or more days per week:	
over the first year	0.78 (0.32 to 1.89)
over the first three months	0.88 (0.50 to 1.56)

The adjusted odds ratio based on randomised allocation (i.e. intention to treat analysis) is 0.94 (95% CI 0.72 to 1.22). 1210 participants included in all models above.

Table S18: Subgroup analyses for primary outcome of eczema between the age of 1 and 2 years

	Intervention	Control	Adjusted interaction effect (relative risk) (95% CI)	Adjusted interaction effect (risk difference) (95% CI)
**Number of first degree relatives with atopic disease**				
1	51/223 (23%)	46/223 (21%)		
2	53/252 (21%)	65/259 (25%)	0.76 (0.47 to 1.22)	-6.3% (-16.9% to 4.3%)
3 or more	35/123 (28%)	39/130 (30%)	0.86 (0.51 to 1.44)	-3.8% (-17.4% to 9.8%)
**Number of first degree relatives with history of eczema**				
0	19/110 (17%)	16/100 (16%)		
1	69/279 (25%)	75/310 (24%)	0.96 (0.49 to 1.88)	-0.5% (-12.7% to 11.7%)
2 or more	51/209 (24%)	59/202 (29%)	0.78 (0.39 to 1.55)	-5.9% (-19.0% to 7.3%)
***FLG* genotype for children with mother and father of white ethnicity and children of other ethnicity with mutation[1]**				
+/+ (no mutations)	66/339 (19%)	79/352 (22%)		
+/- (one *FLG* null mutation)	22/62 (35%)	20/60 (33%)	1.20	5.1%
-/- (two *FLG* null mutations)	1/1 (100%)	1/2 (50%)	(0.70 to 2.09)	(-12.6% to 22.9%)
**Season of birth**				
Spring	47/183 (26%)	38/169 (22%)		
Summer	43/172 (25%)	51/185 (28%)	0.82 (0.49 to 1.37)	-6.1% (-18.8% to 6.6%)
Autumn	30/137 (22%)	35/156 (22%)	0.87 (0.49 to 1.54)	-4.0% (-17.0% to 9.0%)
Winter	19/106 (18%)	26/102 (25%)	0.62 (0.32 to 1.18)	-11.0% (-25.2% to 3.1%)
**Water hardness**				
Soft/moderate	66/270 (24%)	60/274 (22%)		
Hard/very hard	71/323 (22%)	90/334 (27%)	0.76 (0.50 to 1.14)	-7.0% (-16.6% to 2.6%)
**Parental report of regular use of probiotic supplements during pregnancy**				
No	107/457 (23%)	121/463 (26%)		
Yes	9/31 (29%)	9/30 (30%)	1.01 (0.45 to 2.27)	0% (-23.6% to 23.5%)

1 - *Two groups for FLG genotype used in model including interaction effect: +/+ (no mutations) and +/- or -/- (one or two FLG null mutations) due to the small number of participants with two FLG null mutations.*

p-values for interaction effect between subgroup and allocated group (using model for relative risk, n = 1210 unless other stated): 0.51 for number of first degree relatives with atopic disease, 0.58 for number of first degree relatives with history of eczema, 0.51 for FLG genotype in two categories (n = 816), 0.53 for season of birth, 0.18 for water hardness (n = 1201) and 0.98 for use of probiotic supplement during pregnancy (n = 981).

Figure S3: Forest plot of adjusted relative risk for primary outcome with 95% confidence intervals in each subgroup

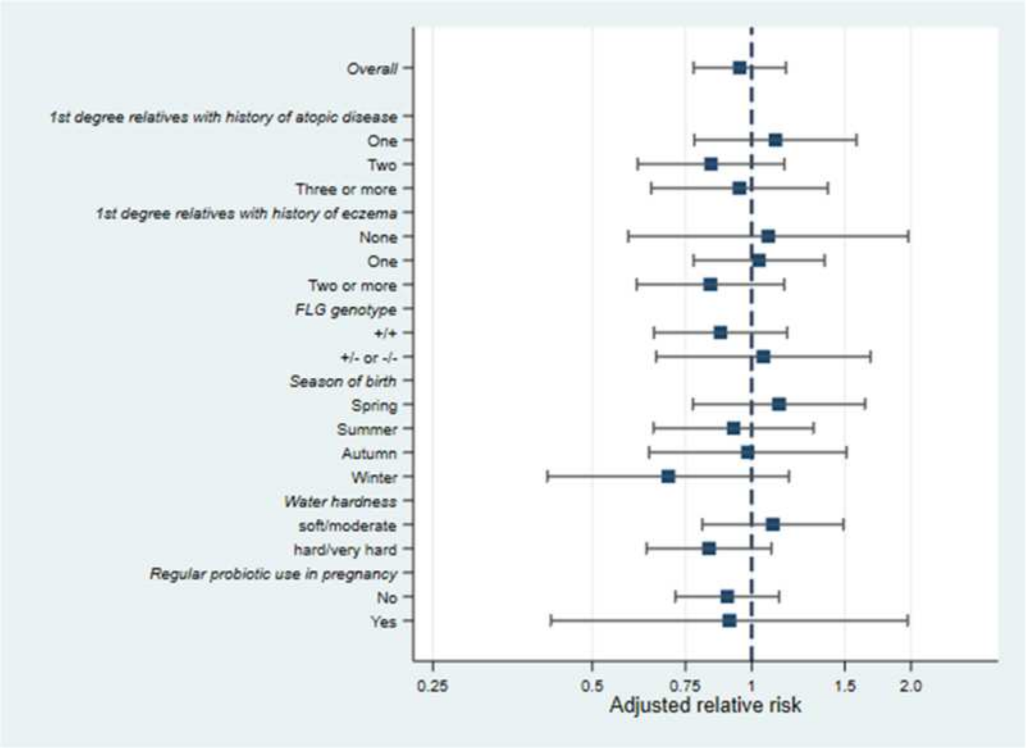

Figure S4: Forest plot of adjusted risk difference for primary outcome with 95% confidence intervals in each subgroup

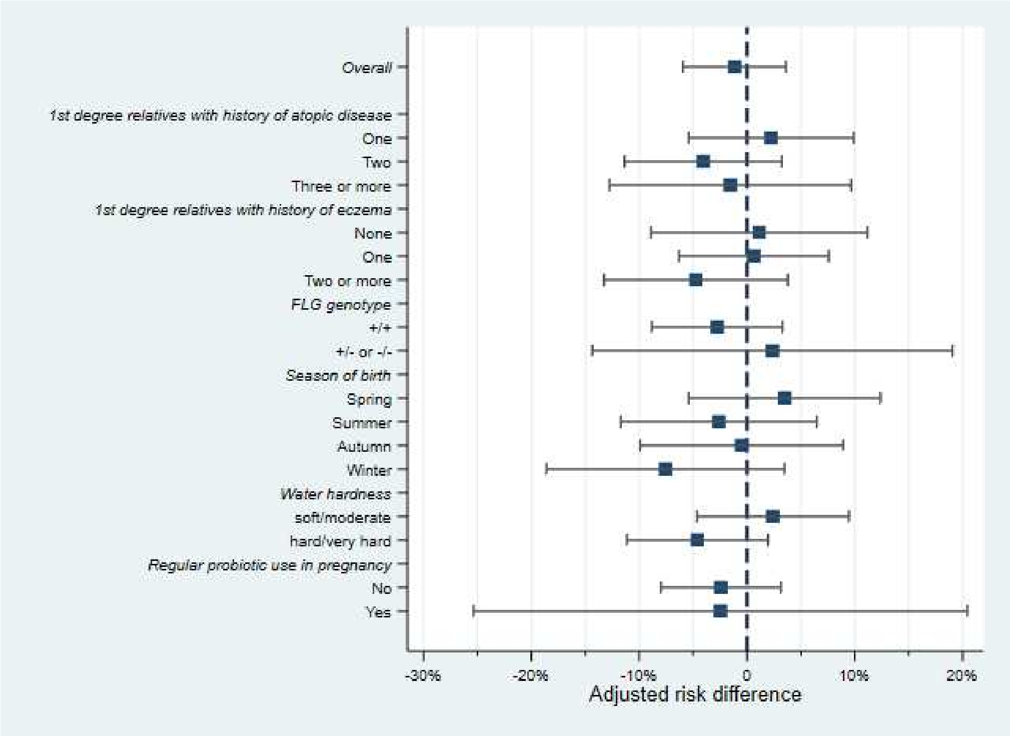

Table S19: Eczema severity (POEM and EASI)

a) Eczema Area and Severity Index (EASI)

		Intervention (n = 553)	Control (n = 567)
Severity of eczema[1] – n (%)			
	Clear	355 (64%)	357 (63%)
	Almost clear	112 (20%)	125 (22%)
	Mild	77 (14%)	75 (13%)
	Moderate	9 (2%)	10 (2%)
	Severe	-	-
	Very severe	-	-
Summary statistics			
	Mean [sd]	0.6 [1.8]	0.6 [1.7]
	Median [25th, 75th centile]	0 [0, 0.5]	0 [0, 0.5]
	Min, max	0, 20.5	0, 16.4
	n	553	567

EASI assessed by research nurse at face-to-face visit. EASI scores range from 0 to 72 with higher scores indicating more severe eczema with a minimal clinically important difference of 6.6 points [4]

b) Patient Orientated Eczema Measure (POEM)

		Intervention	Control
**12 months**			
Severity of eczema[1] – n (%)			
	Clear/almost clear	409 (80%)	414 (79%)
	Mild	51 (10%)	59 (11%)
	Moderate	44 (9%)	42 (8%)
	Severe	7 (1%)	6 (1%)
	Very severe	1 (<0.5%)	1 (<0.5%)
Summary statistics			
	Mean [sd]	1.8 [4.1]	1.7 [3.8]
	Median [25th, 75th centile]	0 [0, 1]	0 [0, 1]
	Min, max	0, 28	0, 26
	n	512	522
**24 months**			
Severity of eczema[1] – n (%)			
	Clear/almost clear	451 (78%)	466 (78%)
	Mild	67 (12%)	78 (13%)
	Moderate	50 (9%)	42 (7%)
	Severe	7 (1%)	9 (2%)
	Very severe	1 (<0.5%)	-
Summary statistics			
	Mean [sd]	1.9 [3.9]	1.9 [4.0]
	Median [25th, 75th centile]	0 [0, 2]	0 [0, 2]
	Min, max	0, 26	0, 24
	n	576	595

POEM scores range from 0 to 28 with higher scores indicating more severe eczema with a minimal clinically important difference of between 3and 3.9 points. [4,5]

Table S20: Time to onset of eczema based on first parental report of clinical diagnosis of eczema

	First parental report of a clinical diagnosis of eczema[1]		Total with parental report of a clinical diagnosis of eczema[2]	
	Intervention	Control	Intervention	Control
3 months	60/534 (11%)	60/518 (12%)	60/594 (10%)	60/584 (10%)
6 months	73/477 (15%)	75/462 (16%)	133/591 (23%)	135/578 (23%)
12 months	61/406 (15%)	67/409 (16%)	194/594 (33%)	202/596 (34%)
18 months	25/335 (7%)	29/325 (9%)	219/588 (37%)	231/593 (39%)
24 months	24/391 (6%)	26/385 (7%)	243/594 (41%)	257/601 (43%)

1 – *numerator is number of children with first parental report of clinical diagnosis of eczema at time point. The denominator is the number of children where questionnaire completed at time point i with no clinical diagnosis of eczema reported at a previous time point.*

2 – *numerator is the number of children with first parental report of a clinical diagnosis of eczema at or before time point i. The denominator is number of children with parental report of a clinical diagnosis of eczema at or before time point i + children whose parents responded that the child had never had a diagnosis of eczema at 24 months + children whose parents responded as no about clinical diagnoses of eczema on all questionnaires up to and including timepoint i (if 24 month follow-up not completed).*

Figure S5: Time to onset of eczema based on first parental report of a clinical diagnosis of eczema

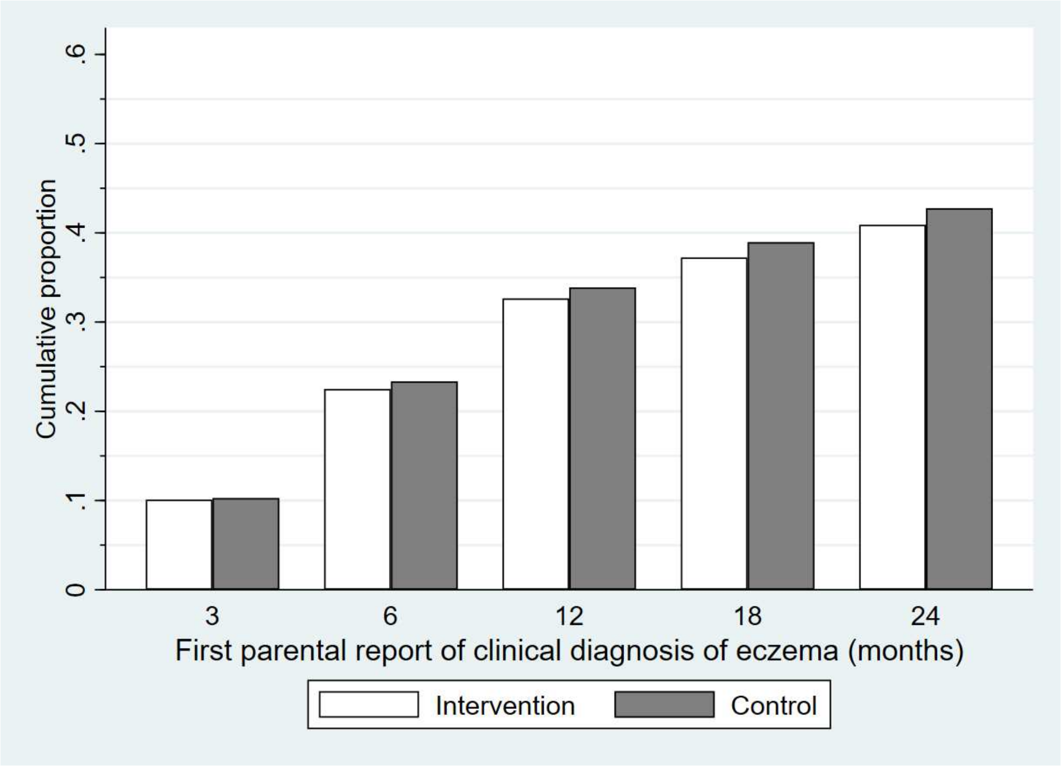

Table S21: Time to onset of eczema based on first parental report of a topical corticosteroid or immunosuppresant prescription for eczema AND a parental report of a clinical diagnosis of eczema.

	First parental report of a topical corticosteroid and/or immunosuppressant prescription for eczema [1]		*Total with parental report of a topical corticosteroid and/or immunosuppressant prescription for eczema* [2]	
	Intervention	Control	Intervention	Control
3 months	25/534 (5%)	11/518 (2%)	25/594 (4%)	11/584 (2%)
6 months	46/510 (9%)	45/508 (9%)	71/583 (12%)	56/568 (10%)
12 months	43/455 (9%)	39/480 (8%)	114/571 (20%)	95/574 (17%)
18 months	24/397 (6%)	24/428 (6%)	138/562 (25%)	119/566 (21%)
24 months	27/447 (6%)	29/484 (6%)	165/568 (29%)	148/573 (26%)

1 – *numerator is number of children with first parental report of a topical corticosteroid and/or immunosuppressant prescription for eczema at time point i (and a parental report of a clinical diagnosis of eczema at or before time point i), denominator is number of children where questionnaire completed at time point i with no topical corticosteroid and/or immunosuppressant prescription for eczema reported at a previous time point*

2– *numerator is the number of children with a parental report of a topical corticosteroid and/or immunosuppressant prescription for eczema before or at time point i (and a parental report of a clinical diagnosis of eczema at or before the time point where topical corticosteroid and/or immunosuppressant prescription for eczema reported). The denominator is the numerator + children with no parental report of a clinical diagnosis of eczema by time point i (derived as table 30) + children with a parental report of a clinical diagnosis of eczema by time point i whose parents did not report a topical corticosteroid and/or immunosuppressant prescription for eczema on all questionnaires up to and including time point i.*

Figure S6: Time to onset of eczema based on first parental report of a topical corticosteroid and/or immunosuppressant prescription for eczema AND a parental report of a clinical diagnosis of eczema.

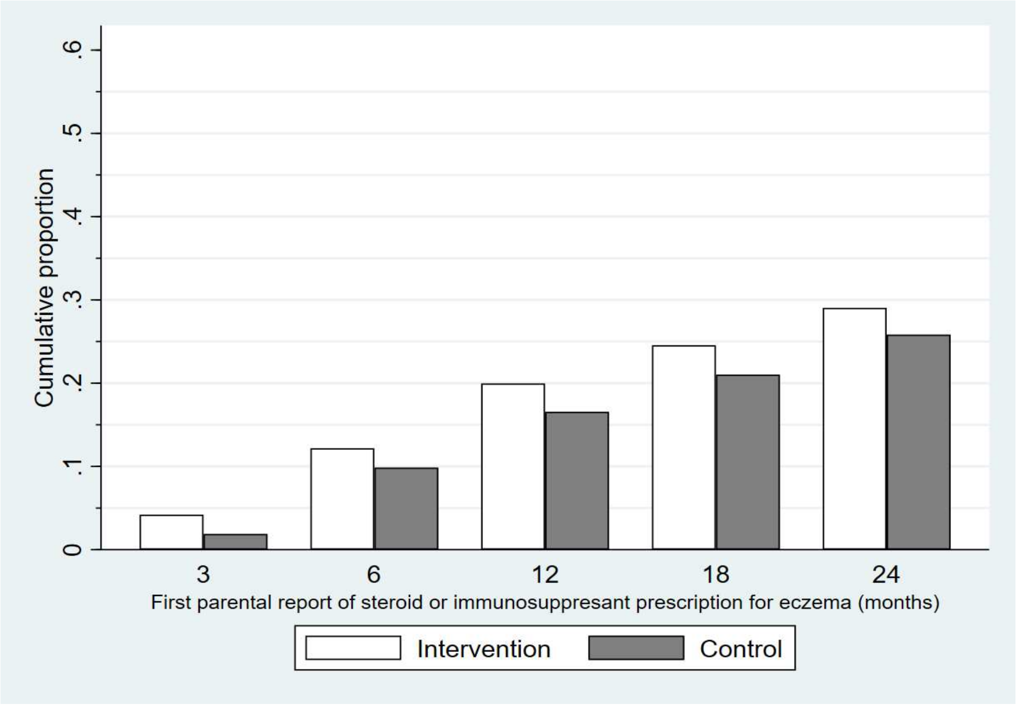

Table S22: Confirmed food allergy at 2 years

	Intervention	Control
**Cow's milk**		
Not allergic based on parental report and/or SPT	544 (94%)	570 (95%)
Not allergic confirmed by OFC	4 (1%)	1 (<0.5%)
Allergic confirmed by OFC	1 (<0.5%)	-
Allergic by panel consensus	8 (1%)	8 (1%)
Not allergic by panel consensus	14 (2%)	14 (2%)
Unclear – possible food allergy	-	1 (<0.5%)
Unclear – food allergy unlikely	5 (1%)	4 (1%)
n	576	598
**Egg**		
Not allergic based on parental report and/or SPT	493 (86%)	525 (88%)
Not allergic confirmed by OFC	12 (2%)	8 (1%)
Allergic confirmed by OFC	12 (2%)	3 (1%)
Allergic by panel consensus	21 (4%)	19 (3%)
Not allergic by panel consensus	22 (4%)	26 (4%)
Unclear – possible food allergy	6 (1%)	3 (1%)
Unclear – food allergy unlikely	10 (2%)	14 (2%)
n	576	598
**Peanut**		
Not allergic based on parental report and/or SPT	500 (87%)	513 (86%)
Not allergic confirmed by OFC	16 (3%)	16 (3%)
Allergic confirmed by OFC	6 (1%)	3 (1%)
Allergic by panel consensus	4 (1%)	5 (1%)
Not allergic by panel consensus	29 (5%)	35 (6%)
Unclear – possible food allergy	3 (1%)	4 (1%)
Unclear – food allergy unlikely	18 (3%)	22 (4%)
n	576	598
**Overall classification**		
OFC allergic	15 (3%)	6 (1%)
Allergic by panel consensus	26 (5%)	23 (4%)
Panel consensus possible allergy	6 (1%)	2 (<0.5%)
Passed OFC	24 (4%)	15 (3%)
Panel consensus allergy unlikely	23 (4%)	28 (5%)
Not allergic by panel consensus	39 (7%)	51 (9%)
Not allergic based on parental report and/or SPT	443 (77%)	473 (79%)
total	576	598

SPT = skin prick test
OFC = oral food challenge

Table S23: Sensitivity analysis for confirmed food allergy at 2 years, including panel consensus decisions of unclear (food allergy possible or food allergy unlikely)

	Intervention (n = 576)	Control (n = 598)	Adjusted relative risk (95% CI)	Adjusted difference in risk (95% CI)
**Allergic to cow's milk**[1]	9 (1.6%)	9 (1.5%)	1.04 (95% CI 0.42 to 2.60)	0.1% (95% CI -1.3% to 1.5%)
**Allergic to egg**	39 (6.8%)	25 (4.2%)	1.63 (95% CI 1.00 to 2.65)	2.5% (95% CI -0.1% to 5.1%)
**Allergic to peanut**	13 (2.3%)	12 (2.0%)	1.12 (95% CI 0.51 to 2.45)	0.3% (95% CI -1.3% to 2.0%)
**Allergic to at least one of milk, egg or peanut**	47 (8.2%)	31 (5.2%)	1.59 (95% CI 1.02 to 2.46)	2.9% (95% CI 0.0% to 5.7%)

1 - *unadjusted relative risk and difference in risk reported for cow's milk. The model including stratification variables did not converge.*

Table S24: Sensitivity analysis for confirmed food allergy at 24 months to any of milk, egg or peanut using multiple imputation for missing outcomes

	Intervention (n = 693)	Control (n = 701)	Adjusted relative risk (95% CI)	Adjusted difference in risk (95% CI)
**Allergic to at least one of milk, egg or peanut**				
Using multiple imputation model as specified in SAP	9.6% (SE 1.4%)	6.5% (SE 1.2%)	1.49 (0.94 to 2.36)	3.1% (-0.5% to 6.7%)
Using multiple imputation model with randomisation stratification variables only	7.6% (SE 1.1%)	5.1% (SE 1.0%)	1.49 (0.93 to 2.36)	2.5% (-0.3% to 5.3%)

Table S25: Summary of compliance/contamination and confirmed food allergy at 24 months to any of milk, egg or peanut

	**Allergic to at least one of milk, egg or peanut**	
**Level of compliance/contamination**	Intervention n (%)	Control n (%)
Participants with complete data on compliance/contamination		
Full	11/291 (4%)	4/51 (8%)
Early onset application	13/79 (16%)	0/35 (0%)
Late onset application	1/14 (7%)	8/77 (10%)
None	2/25 (8%)	5/242 (2%)
All participants with confirmed food allergy diagnosis available		
Full	16/322 (5%)	8/68 (12%)
Early onset application	17/125 (14%)	2/51 (4%)
Late onset application	1/18 (6%)	8/81 (10%)
None	7/82 (9%)	11/368 (3%)

Table S26: Complier average causal effect for confirmed food allergy at 2 years to any of milk, egg or peanut

	Adjusted odds ratio (95% CI)
Compliance defined as widespread emollient use over the child's body at least three or more days per week:	
over the first year	see footnote[1]
over the first three months	1.79 (0.62 to 5.14)

Note adjusted odds ratio based on randomised allocation (i.e. intention to treat analysis) is 1.51 (95% CI 0.92 to 2.47). 1115 participants included in all models above.

1 – *latent growth class mixture model gives odds ratio of 11.9 with lower limit of 95% confidence interval of 0 and upper limit of* $e^{34.69}$*, suggesting problem with the estimation procedure*

Table S27: Subgroup analyses for confirmed food allergy at 2 years to any of milk, egg or peanut

	Intervention	Control	Adjusted interaction effect (relative risk) (95% CI)	Adjusted interaction effect (risk difference) (95% CI)
**Number of first degree relatives with atopic disease**				
1	14/207 (7%)	9/208 (4%)		
2	17/234 (7%)	14/244 (6%)	0.81 (0.28 to 2.35)	-0.9% (-7.2% to 5.3%)
3 or more	10/106 (9%)	6/116 (5%)	1.21 (0.44 to 3.31)	1.8% (-6.4% to 10.0%)
**Number of first degree relatives with history of eczema**				
0	6/103 (6%)	2/95 (2%)		
1	21/255 (8%)	12/289 (4%)	0.73 (0.13 to 4.06)	0.4% (-6.3% to 7.1%)
2 or more	14/189 (7%)	15/184 (8%)	0.33 (0.06 to 1.86)	-4.3% (-12.0% to 3.3%)
***FLG* genotype for children with mother and father of white ethnicity and children of other ethnicity with mutation[1,2]**				
+/+ (no mutations)	14/325 (4%)	12/344 (3%)		
+/- (one *FLG* null mutation)	8/59 (14%)	4/56 (7%)	1.57	5.6%
-/- (two *FLG* null mutations)	0/1 (0%)	0/2 (0%)	(0.40 to 6.17)	(-5.6% to 16.8%)

1 - *Two groups for FLG genotype used in model including interaction effect: +/+ (no mutations) and +/- or -/- (one or two FLG null mutations) due to the small number of participants with two FLG null mutations.*
2 - *Unadjusted estimates are reported for FLG genotype as the model including stratification variables did not converge.*

p-values for interaction effect between subgroup and allocated group: 0.83 for number of first degree relatives with atopic disease (n= 1115), 0.21 for number of first degree relatives with history of eczema (n = 1115) and 0.52 for FLG genotype in two categories (n = 787).

Figure S7: Forest plot of adjusted relative risk for confirmed food allergy at 24 months to any of milk, egg or peanut with 95% confidence intervals in each subgroup

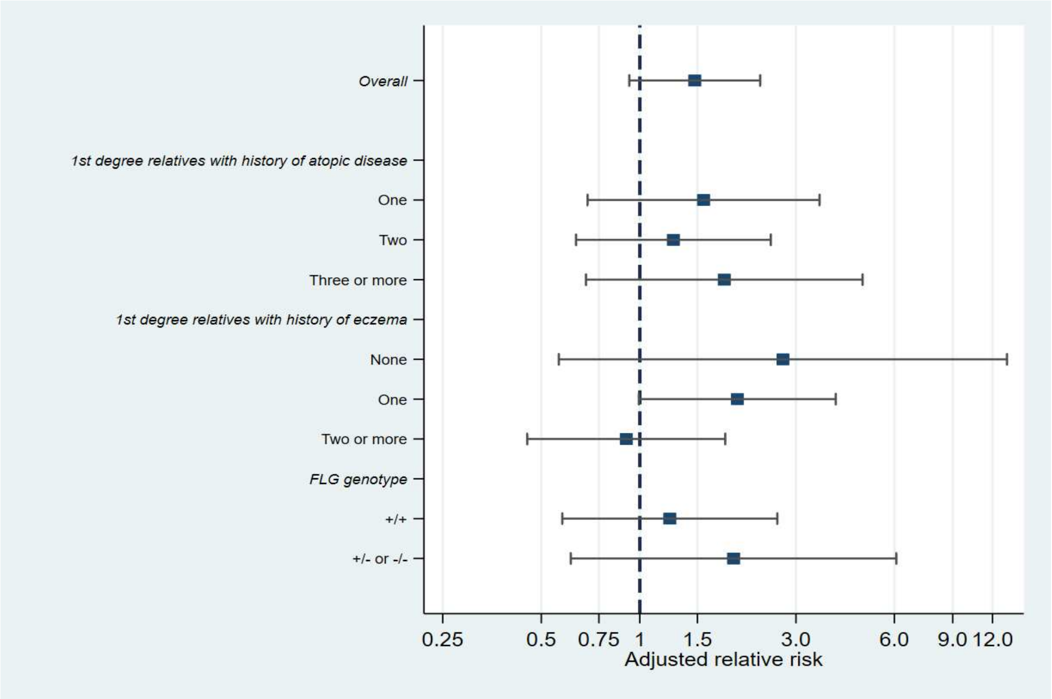

Unadjusted estimates shown for *FLG* genotype as model with stratification variables did not converge

Figure S8: Forest plot of adjusted risk difference for confirmed food allergy at 24 months to any of milk, egg or peanut with 95% confidence intervals in each subgroup

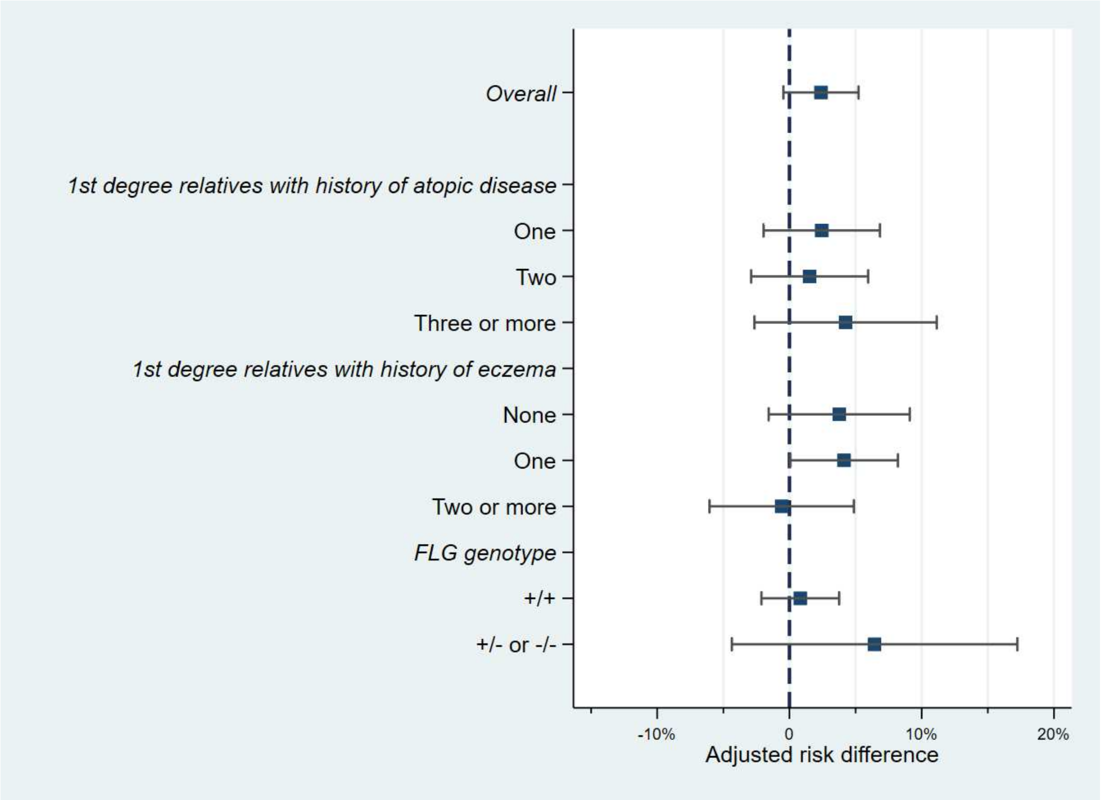

Note unadjusted estimates shown for *FLG* genotype as model with stratification variables did not converge

Table S28: Rate of allergic sensitisation at 2 years

SPT longest wheal diameter	Intervention	Control	Adjusted relative risk of SPT ≥ 3mm (95% CI)	Adjusted difference in risk of SPT ≥ 3mm (95% CI)
Cow's milk				
0mm	461 (94%)	468 (94%)		
1 to 2mm	13 (3%)	19 (4%)		
3 to 6mm	8 (2%)	6 (1%)		
≥ 7mm	6 (1%)	5 (1%)		
n	488	498		
Median [25th, 75th centile]	3 [2, 6]	2 [2, 3]		
Min, max	1, 20	1, 11		
n	27	30		
Egg				
0mm	435 (89%)	450 (90%)		
1 to 2mm	12 (2%)	16 (3%)		
3 to 6mm	20 (4%)	17 (3%)		
≥ 7mm	23 (5%)	16 (3%)		
n	490	499		
Median [25th, 75th centile]	5 [3, 10]	4 [2, 8]		
Min, max	1, 27	1, 18		
n	55	49		
Peanut				
0mm	457 (93%)	468 (93%)		
1 to 2mm	15 (3%)	18 (4%)		
3 to 6mm	17 (3%)	15 (3%)		
≥ 7mm	1 (<0.5%)	1 (<0.5%)		
n	490	502		
Median [25th, 75th centile]	3 [2, 4]	2 [2, 4]		
Min, max	1, 10	1, 7		
n	33	34		
Grass pollen				
0mm	464 (94%)	471 (94%)		
1 to 2mm	18 (4%)	18 (4%)		
3 to 6mm	10 (2%)	13 (3%)		
≥ 7mm	-	-		
n	492	502		
Median [25th, 75th centile]	2 [2, 3]	2 [2, 3]		
Min, max	1, 6	1, 6		
n	28	31		
Cat				
0mm	466 (95%)	471 (94%)		
1 to 2mm	14 (3%)	16 (3%)		
3 to 6mm	11 (2%)	13 (3%)		
≥ 7mm	1 (<0.5%)	-		
n	492	500		
Median [25th, 75th centile]	2 [2, 4]	2 [2, 4]		
Min, max	1, 9	1, 5		
n	26	29		
Dust mite				
0mm	432 (88%)	444 (89%)		
1 to 2mm	27 (5%)	26 (5%)		
3 to 6mm	29 (6%)	26 (5%)		
≥ 7mm	5 (1%)	4 (1%)		
n	493	500		
Median [25th, 75th centile]	3 [2, 4]	3 [2, 4.5]		
Min, max	1, 7	1, 8		
n	61	56		
SPT ≥ 3mm to any allergen	88/490 (18%)	74/498 (15%)	1.22 (0.92 to 1.62)	3.1% (-1.5% to 7.7%)

Table S29: Parent reported food allergy

a) 12 months

	Intervention	Control	Adjusted relative risk (95% CI)	Adjusted difference in risk (95% CI)
**Parental report of clinical diagnosis of food allergy**	54/507 (11%)	44/522 (8%)	1.27 (0.87 to 1.85)	2.4% (-1.1% to 6.0%)
**Parental report of any food allergy**	110/505 (22%)	103/523 (20%)	1.11 (0.87 to 1.40)	2.1% (-2.8% to 7.1%)
reaction to cow's milk	72/508 (14%)	66/525 (13%)		
reaction to egg	45/507 (9%)	32/525 (6%)w		
reaction to nuts	14/503 (3%)	8/522 (2%)		
reaction to other food	40/507 (8%)	40/525 (8%)		
**Parental report of allergy to cow's milk, egg or nut at 12 months**	98/505 (19%)	86/523 (16%)	1.18 (0.91 to 1.53)	3.1% (-1.6% to 7.8%)

b) 24 months

	Intervention	Control	Adjusted relative risk (95% CI)	Adjusted difference in risk (95% CI)
**Parental report of clinical diagnosis of food allergy between 12 and 24 months**	51/573 (9%)	41/598 (7%)		
**Parental report of clinical diagnosis of food allergy at 24 months[1]**				
No	421 (73%)	436 (73%)		
Yes	72 (13%)	66 (11%)	1.12 (0.82 to 1.52)	1.5% (-2.8% to 5.7%)
No diagnosis of food allergy reported between 12 & 24 months, not known between birth and 12 months	82 (14%)	97 (16%)		
n	575	599		
**Parental report of any food allergy at 24 months**	208/574 (36%)	197/597 (33%)	1.10 (0.94 to 1.28)	3.3% (-2.1% to 8.8%)
reaction to cow's milk	86/575 (15%)	77/598 (13%)		
reaction to egg	45/575 (8%)	45/598 (8%)		
reaction to peanut	9/574 (2%)	10/598 (2%)		
reaction to nuts other than peanut	5/574 (1%)	5/598 (1%)		
reaction to other food	141/575 (25%)	124/598 (21%)		
**Total number of times child reacted to any food at 24 months**				
None	367 (64%)	400 (67%)		
One	25 (4%)	38 (6%)		
Two	24 (4%)	18 (3%)		
More than two	159 (28%)	142 (24%)		
n	575	598		
**Parental report of allergy to cow's milk, egg or nut at 24 months**	121/574 (21%)	116/597 (19%)	1.09 (0.87 to 1.36)	1.8% (-2.8% to 6.4%)
**Parental report of immediate food allergy to common allergen at 24 months[2, 3]**	118/574 (21%)	96/597 (16%)	1.28 (1.00 to 1.63)	4.7% (0.2% to 9.1%)
reaction to cow's milk	61/575 (11%)	46/598 (8%)		
reaction to egg	44/575 (8%)	41/598 (7%)		
reaction to peanut	8/574 (1%)	10/598 (2%)		
reaction to nuts other than peanut	4 /574 (1%)	5/597 (1%)		
reaction to other common food allergen[2]	35/575 (6%)	26/598 (4%)		
**Parental report of immediate allergy to cow's milk, egg or peanut at 24 months[2]**	98/574 (17%)	83/598 (14%)	1.23 (0.94 to 1.61)	3.3% (-0.9% to 7.4%)

1 – *995 participants included in analysis model for parent report of clinical diagnosis of food allergy at 24 months. Participants with no diagnosis of food allergy between 12 and 24 months and unknown information between birth and 12 months not included.*
2 – *Common food allergens: cow's milk, egg, peanut, other nuts, fish, sesame, wheat, soya and kiwi fruit.*
3 – *Immediate food allergy defined as reaction within 2 hours of eating the food*

Table S30: Sensitivity analysis for parental report of clinical diagnosis of food allergy at 2 years

	Intervention	Control	Adjusted relative risk (95% CI)	Adjusted difference in risk (95% CI)
**Parental report of clinical diagnosis of food allergy at 24 months**				
No	421 (73%)	436 (73%)		
Yes	72 (13%)	66 (11%)		
No at 24, not known at 12	82 (14%)	97 (16%)		
n	575	599		
**Diagnosis of food allergy between 12 and 24 months ascertained from GP records** for participants with missing data on parental report of a clinical diagnosis at 24 months				
No	17 (89%)	20 (95%)		
Yes	1 (5%)	1 (5%)		
Uncertain/possible	1 (5%)	-		
n	19	21		
**Parental (or GP) report of clinical diagnosis of food allergy at 24 months using multiple imputation** for missing parental report of clinical diagnosis of food allergy between birth and 12 months[1]				
	13.4% (SE 1.4%) (n = 693)	11.5% (SE 1.4%) (n = 701)	1.17 (0.86 to 1.61)	1.9% (-2.0% to 5.8%)

1 – *Variables included in the multiple imputation are: parental report of doctor diagnosis of food allergy between birth and 12 months, parental (or GP) report of doctor diagnosis of food allergy between 12 and 24 months, centre, number of immediate family members with atopic disease and allocated group. Note augmented regression was used for the imputation of doctor diagnosis of food allergy between birth and 12 months and 12 and 24 months in the presence of perfect prediction. Analysis assumes that missing outcome data are missing at random and depend on the observed variables included in the imputation model but not the unobserved outcomes. Thirty datasets were imputed and estimates were combined using Rubin's rules.*

Table S31: Parent-reported skin infections during the first year (as randomised)

	Intervention (n = 585)	Control (n = 589)	Adjusted incidence rate ratio (95% CI)
Number of skin infections reported per child			
Mean [sd]	0.23 [0.68]	0.15 [0.46]	1.55
Median [25th, 75th centile]	0 [0, 0]	0 [0, 0]	(95% CI 1.15 to 2.09)
Min, Max	0, 9	0, 5	
No infections reported	496 (85%)	522 (89%)	
Single infection reported	63 (11%)	52 (9%)	
More than one infection reported	26 (4%)	15 (3%)	
Type of skin infection *Child had at least one infection of:*			
Impetigo	12 (2%)	7 (1%)	
Folliculitis	2 (<0.5%)	2 (<0.5%)	
Boils	1 (<0.5%)	2 (<0.5%)	
Other bacterial infection	10 (2%)	12 (2%)	
Other viral infection	19 (3%)	9 (2%)	
Other fungal infection	26 (4%)	14 (2%)	
Other infection	7 (1%)	5 (1%)	
Didn't know	26 (4%)	20 (3%)	
Did not specify	1 (<0.5%)	1 (<0.5%)	

Based on parental reports of skin infections on the 3, 6 and 12 month questionnaires. The denominator is children with information provided about skin infections on a least one of the 3, 6 or 12 month questionnaires.

Table S32: Infant slippage incidents within an hour of applying skin care products to the baby's skin during the first year (as randomised)

	Intervention (n = 584)	Control (n = 584)	Adjusted risk ratio (95% CI)	Adjusted risk difference (95% CI)
At least one infant slippage incident within an hour of applying skin care products to the baby's skin	15 (3%)	11 (2%)	1.37 (95% CI 0.63 to 2.97)	0.8% (95% CI -0.9% to 2.5%)
Number of questionnaires slippage incidents reported				
One	14 (2%)	11 (2%)		
Two	1 (<0.5%)	-		
Three	-	-		

Slippage incidents include slippages in hand and slippages based on parental reports on the 3, 6 and 12 month questionnaires
The denominator is children with information provided about slippages on a least one of the 3, 6 or 12 month questionnaires.

References

1) Dunn, G., M. Maracy and B. Tomenson (2005). "Estimating treatment effects from randomized clinical trials with noncompliance and loss to follow-up: the role of instrumental variable methods." Statistical Methods in Medical Research 14(4): 369-395.

2) Shrier, I., R. J. Steele, E. Verhagen, R. Herbert, C. A. Riddell and J. S. Kaufman (2014). "Beyond intention to treat: What is the right question?" Clinical Trials 11(1): 28-37.

3) White, I. R., P. Royston and A. M. Wood (2011). "Multiple imputation using chained equations: Issues and guidance for practice." Statistics in Medicine 30(4): 377-399.

4) Schram ME, Spuls PI, Leeflang MM, Lindeboom R, Bos JD, Schmitt J. EASI, (objective) SCORAD and POEM for atopic eczema: responsiveness and minimal clinically important difference. Allergy. 2012 Jan;67(1):99-106. doi: 10.1111/j.1398-9995.2011.02719.x. Epub 2011 Sep 27.

5) Howells L, Ratib S, Chalmers JR, Bradshaw L, Thomas KS; CLOTHES trial team. How should minimally important change scores for the Patient-Oriented Eczema Measure be interpreted? A validation using varied methods. Br J Dermatol. 2018 May;178(5):1135-1142. doi: 10.1111/bjd.16367. Epub 2018 Apr 17.
